# Sustainable improvement of single cross maize performance using vinasse-based biofertilizer

**DOI:** 10.1038/s41598-026-49182-y

**Published:** 2026-05-13

**Authors:** Asmaa A. Mohamed, Adel R. A. Usman, Mohsen A. Gameh, Atif Abo-Elwafa, Bahy R. Bakheit

**Affiliations:** 1 Department of Agronomy, Faculty of Agriculture, Assiut University, Assiut, 71526 Egypt; 2 Department of Soils and Water, Faculty of Agriculture, Assiut University, Assiut, 71526 Egypt

**Keywords:** Single crosses maize, Fertilization with vinasse, Correlation, AMMI analysis, GGE biplot analysis, Ecology, Ecology, Plant sciences

## Abstract

Sustainable maize (*Zea mays* L.) production can be enhanced through effective biofertilization practices, such as the application of vinasse, which improves plant growth and yield traits. This study aimed to evaluate the effect of vinasse as an organic biofertilizer on the growth, yield, and quality of three maize single crosses, and to assess genotype–trait relationships using GGE biplot analysis for sustainable single-cross selection. A two-year field experiment was conducted during the 2022 and 2023 seasons at the Agronomy Experimental Farm, Faculty of Agriculture, Assiut University, Egypt. The experiment was arranged in a strip-plot design within a randomized complete block design (RCBD) with three replications. Treatments included four vinasse solution levels (0, 1, 2, and 3 L plot^−1^; 10.5 m^2^ and three maize single crosses (SC2031, SC2036, and SC168). The results revealed significant effects of vinasse concentrations, single-cross genotypes, and their interaction on major agronomic traits. The highest grain yield per plant was obtained from the single cross SC2036 under the application of 3 L plot^−1^ of vinasse, recording 187.0 and 191.2 g in the first and second seasons, respectively, corresponding to a maximum grain yield of 8.60 t ha^−1^. The single cross SC2036 showed superior performance in plant height, leaf number, flag leaf area, chlorophyll content, stem and ear characteristics, grain yield, 100-grain weight, protein, and oil content. Meanwhile, SC2031 exhibited the highest yield stability across seasons, followed by SC2036 and SC168. AMMI analysis confirmed strong genotype × treatment interactions, with more than 98% of the total variation explained by the first two IPCA axes, indicating that IPCA1 was the major contributor to the interaction effect. These findings highlight the promising role of vinasse application combined with appropriate single-cross selection in improving maize productivity and supporting sustainable maize production under Egyptian agro-ecological conditions.

**Supplementary Information:**

The online version contains supplementary material available at 10.1038/s41598-026-49182-y.

## Introduction

Maize (*Zea mays* L.) belongs to the family Poaceae and is one of the most important cereal crops worldwide^1^. It has multiple uses in food, industry, and bioenergy^2^ and ranks third after wheat and rice in global production^3^. In addition, maize is valued for its high nutritional content, including energy, minerals, vitamins, and essential amino acids^4^. The crop residues and stalks are widely used for silage production, providing an important feed source for livestock and contributing to farmers’ income^5^. In Egypt, maize is cultivated on approximately 950,000 hectares, producing about 7.13 million tons of grain annually^6^. However, maize productivity is strongly influenced by fertilization practices, particularly nitrogen and phosphorus management. The efficiency of these fertilizers varies depending on the cultivar and environmental conditions, as genetic differences among maize genotypes affect nutrient uptake and utilization under different agro-ecological conditions. Furthermore, the increasing cost and limited availability of chemical fertilizers have become major challenges for sustainable maize production, highlighting the need for alternative nutrient sources.

The selection of a maize cultivar is one of the most important factors influencing both the quantity and quality of yield^7^. Significant production gains reflected in increased yield can be achieved without incurring additional costs. Recent advances in breeding have led not only to the development of numerous new single cross but also to shifts in the selection of breeding types^8^ Consequently, components of grain yield structure, such as the number of productive ears, the weight of 1000 grains, and the number of grains per ear can influence overall yield to varying degrees, depending on the single cross type.

Vinasse is a liquid byproduct of the fermentation process used to produce alcohol from raw materials. It is commonly utilized in agriculture as a low-cost nutrient supplement^9,10^. Vinasse contains high levels of phosphate, sodium, sulfate, potassium, calcium, iron, carbon, essential micronutrients, and organic compounds, making it a valuable resource for improving soil fertility and increasing agricultural productivity when used effectively in irrigation and fertilization practices^11^. Studies have shown that vinasse can improve crop yield and reduce the reliance on mineral fertilizers^12,13^. In terms of grain yield, optimal results were recorded with applications of 150 m^3^ ha^−1^ in 2013 and 2014, and 100 m^3^ ha^−1^ in 2015, each yielding over 12 tons per hectare^14^. Vinasse offers agronomic benefits but can cause salinity and sodium buildup if mismanaged, so careful application rates are crucial in arid regions.

Despite increasing interest in vinasse, its interaction with maize single-crosses and optimal application rates under arid and semi-arid conditions remains unclear. This study evaluated the agronomic performance and yield stability of maize single crosses under different vinasse levels. The Genotype-by-Trait (GT) biplot, derived from the GGE (Genotype and Genotype × Environment) methodology, is a powerful multivariate tool used to evaluate the influence of specific traits on genotypic performance. GT biplots facilitate the exploration of genetic correlations among traits^15,16^ and have been successfully applied in the assessment of various crops, including maize^17–21^. They provide a visual framework for comparing genotypes based on multiple traits and enable the identification of genotypes with desirable trait combinations^16,18,20,22–24^.

This study evaluates vinasse, a byproduct of ethanol production, as an organic fertilizer for the cultivation of single-cross maize hybrids, enriching the soil with essential nutrients such as potassium and enhancing crop growth and yield. Despite extensive research on maize fertilization, there is limited information on the response and stability of these specific single-cross hybrids (SC2031, SC2036, SC168) under varying vinasse levels in semiarid regions like Assiut, Egypt, where soil fertility and water availability pose challenges for sustainable maize production.

Therefore, this research uniquely combines vinasse fertilization at multiple doses with GGE biplot analysis to evaluate trait–genotype interactions in these hybrids, offering a sustainable, data-driven approach to optimize hybrid selection, yield, and stability under local agro-ecological conditions.

## Materials and methods

### Experimental site

The current study was carried out for two summer seasons of 2022 and 2023 at the Experimental Farm of the Agronomy Department, Faculty of Agriculture, Assiut University, Egypt (27° 08′ 20.8″ N, 31° 19′ 40.5″ E). Soil samples were collected at the beginning of each field experiment from the topsoil layer (0–15 cm depth) to assess the initial physicochemical properties of the experimental site, ensuring sample uniformity. Samples were analyzed in the laboratory to determine key soil characteristics, including particle size distribution, pH, EC, CaCO_3_, total N (Table 1) according to Estefan et al.^25^. Table 2 show means of air temperature and relative humidity and precipitation obtained from etiological station at Assiut, Egypt, during the two growing seasons. Climatic data, including mean air temperature, relative humidity, and precipitation during the 2022 and 2023 growing seasons, were obtained from the local meteorological station. The two seasons were characterized by comparable temperature and relative humidity patterns, with no recorded rainfall during the experimental periods. Therefore, crop water requirements were fully supplied through controlled irrigation.

**Table 1:** Some physical and chemical properties of the experimental soil.

Season	2022	2023
Particle size analysis		
Sand	26.00	26.40
Silt	25.50	25.40
Clay	48.50	48.20
Soil texture	Clay	Clay
Chemical analysis		
pH	7.77	7.77
EC_e_	2.11	2.03
Organic matter %	1.72	1.70
Total N%	0.09	0.08
CaCO_3_%	1.17	1.22

**Table 2:** Means of air temperature and relative humidity and precipitation obtained from etiological station at Assiut, Egypt, during the two growing seasons. *Source*: Meteorological authority, Assiut, Egypt; H, relative humidity; P, precipitation, no precipitation at Assiut, Egypt

Month	Temperature (°C)		H (%)	*P* (in)
	Min	Max		
2022				
May	19.1	34.5	27	0
June	22.2	37.2	30	0
July	23.1	37.5	32	0
August	23.8	37.6	39	0
September	22.0	36.4	38	0
2023				
May	21.0	35.5	29	0
June	25.0	38.0	30	0
July	25.0	39.0	33	0
August	24.0	38.0	38	0
September	23.0	37.2	36	0

### Experimental treatments and design

The experiment was conducted using a strip-plot design arranged in a randomized complete block with three replications. Three single-cross maize single cross (SC2031, SC2036, and SC168) were assigned to horizontal strips. The three single crosses were obtained from High Tech Seeds Company during the two growing seasons, whereas four vinasse application levels (0, 1, 2, and 3 L plot^−1^) were randomly allocated to vertical strips. Each replication consisted of all possible combinations of single cross and vinasse levels. The intersection of each horizontal and vertical strip represented an experiment. Resulting in a total of 12 treatment combinations. Each plot measured 3 × 3.6 m (10.8 m^2^ and consisted of five ridges, each 3.6 m long and 60 cm wide. Maize seeds were sown at a spacing of 20 cm between hills, with one seed per hill, planted on one side of each ridge. Sowing dates were June 8, 2022, and June 5, 2023. Fertilization treatments included four levels of vinasse solution plus: The applied rates of a control 0 L/plot (VSL0), 1 L/plot (VSL1), 2 L/plot (VSL2), and 3 L/plot (VSL3) (10.8 m^2^ correspond to approximately (i.e., 0, 92.6, 185.2, and 277.8 m^3^/ha), respectively. Each vinasse dose was diluted in 5 L of distilled water. Vinasse was applied uniformly as a single soil application at the 3–5 leaf stage on moist soil. This growth stage represents a critical early vegetative phase characterized by active root development and high nutrient uptake efficiency in maize. A single application was adopted to ensure controlled nutrient supply, avoid potential salt accumulation associated with repeated vinasse applications, and allow clearer evaluation of genotype × vinasse interaction effects. Application of moist soil facilitated uniform infiltration and improved nutrient availability while minimizing surface losses. All plots received equal amounts of irrigation to avoid confounding effects. Differences among treatments were therefore attributable solely to vinasse concentration.

Six irrigations were applied according to the recommended maize schedule, and all plots received equal amounts of water. Soil moisture was maintained near field capacity. Vinasse was applied since 30 days after planting, onto moist soil to avoid potential root injury and to ensure uniform distribution within the root zone. The agricultural practices for the maize crop, including fertilization, irrigation, weed and disease control, management and harvesting, were carried out as recommended. The recommended nitrogen dose is 280 nitrogen units per hectare in two doses, while phosphorus is added during land preparation for planting at a rate of 72 phosphorus units per hectare. There are 280 nitrogen doses per hectare commended in two doses, while phosphorus is added during land preparation for planting at a rate of 72 phosphorus units per hectare. Indeed, vinasse contains appreciable amounts of nitrogen and phosphorus, and we acknowledge that plots receiving vinasse (particularly the high-dose treatment, VSL3) received more total nutrients compared to the control. We intended to maintain a baseline of recommended N and P fertilization across all plots to ensure comparability with standard farmer practices in Egypt. At the same time, vinasse was evaluated as an additional amendment. The chemical analysis of vinasse from the Abo-Korkas sugarcane factory showed that it is an acidic organic material (pH 4.20) with relatively high electrical conductivity (15.35 Ds m^−1^). Vinasse contained appreciable amounts of organic matter (4.79%) and organic carbon (2.78%), indicating its potential to improve soil fertility. It was rich in macronutrients, particularly potassium (30,000 ppm as K_2_O), in addition to total nitrogen (5900 ppm) and total phosphorus (2300 ppm as P_2_O_5_). Moreover, vinasse supplied essential micronutrients such as iron, manganese, zinc, and copper, while its sodium content (4350 ppm) suggests that application rates should be carefully managed to avoid salinity-related effects (Table 3).

**Table 3:** Chemical analysis of vinasse of Abo-Korkas sugarcane factory at El-Minya; Egypt (2022).

Property	Unit	Value
pH		4.200
EC (dS m^− 1^)	dS m^−1^	15.35
Organic carbon%	%	2.78
Organic matter %	%	4.79
Total N	ppm	5900
Total P (P_2_O_5_)	ppm	2300
Total K (K_2_O)	ppm	30,000
Total Na	ppm	4350
Total Fe	ppm	432.55
Total Mn	ppm	26.00
Total Zn	ppm	32.31
Total Cu	ppm	10.46

#### Measured characters

A. Growth characters:
  Five plants were randomly collected from each plot at maturity and measured as follows:
  1. Plant height, cm (PH): Length of the main stem from soil surface to plant apex has been measured using a ruler.
  2. Number of leaves/ plant (NL).
  3. Flag leaf area (FLA) was calculated using the method of McKee^26^ it involved measuring the length of the leaf blade from its base to the tip of the leaf (leaf L) and the width of the leaf at its widest point (leaf W) and multiplying them by a correction factor (0.75) as illustrated in the equation: Flag leaf area (FLA) = Leaf L × Leaf W × 0.75.
  4. Chlorophyll mg/m^2^ (Chl.):
    Chlorophyll content was determined in the flag leaves of five plants from each plot at 60 days after planting by chlorophyll meter SPAD-502 plus, as reported Dash et al.^27^. SPAD calibration equations:
    $${\mathrm{Y}}\,=\,0.091{\text{ x}}2\,+\,1.594{\mathrm{x}}\,+\,32.41$$
    where Y represents chlorophyll concentration and x represent SPAD value. The calibration equation was used to convert SPAD readings into actual chlorophyll concentration, providing a more quantitative and physiologically meaningful estimate than SPAD units alone. The calibration equation used to convert SPAD meter readings (x) into actual chlorophyll concentration (Y), expressed of leaf area.
  5. Stem diameter/plant cm (SD).
  6. Ear height in meters cm (EL): height from the ground surface to the top-most bearing node.

B. Grain yield and its attributes:
  The harvest was performed when the ears had dry grain characteristics as follows:
  1. Ear diameter cm (ED): Was measured and recorded in centimeter as the thickness of the ear at the middle of the ear.
  2. Ear length cm (EL): Length of the ear was measured and recorded in centimeter from the base to the tip of the ear at the time of harvest.
  3. Grains yield/ plant g (GYP).
  4. 100-grain weight g (SI): Weight of 100-grains was randomly sampled from each treatment.
  5. Shelling percentage (SP).
  6. Grain yield ton ha^−1^(GY): Weight of grain yield of each plot adjusted to 15.5% moisture content were recorded.

C. Grain quality
  1. Protein % (PP). Total nitrogen (N) was determined using the micro Kjeldahl method according to AOAC^28^.
  2. Oil percentage %(OP): oil percentage in grain was extracted by Soxhlet apparatus and petroleum ether (bp 60–80c) as solvent according to AOAC^28^.

### Statistical analysis

Analysis of variance (ANOVA) for data from both seasons was performed following Gomez & Gomez^29^ and mean comparisons were made using the least significant difference (LSD) test at the 5% level^30^. Phenotypic correlation coefficients among traits were calculated using and, based on the method of R Studio^31^. The Additive Main Effects and Multiplicative Interaction (AMMI) model was employed to evaluate the genotype-by-trait (GT) interaction under varying vinasse applications. Unlike the standard application of AMMI for multi-environment trials^32^, this approach utilized the model to assess the stability and performance consistency of several physiological, agronomic, and quality traits across four vinasse levels for each single cross of maize. In addition, trait stability in this study was evaluated based on the coordinates within the AMMI biplot. Since, traits positioned near the biplot origin (zero IPCA scores) were identified as stable^33^, indicating consistent performance across different vinasse levels. Conversely, traits with higher absolute IPCA values demonstrated greater sensitivity to vinasse treatments. To enhance the reliability of the trait stability assessment, the AMMI stability value (ASV) for each trait was calculated based on the relative contributions of the principal component axis scores (IPCA1 and IPCA2) to the interaction sum of squares. The ASV, as proposed by Purchase et al.^34^, was calculated as follows:

$${\mathrm{ASV}}=\sqrt {{{\left( {\frac{{{\mathrm{IPCA}}1{\mathrm{SS}}}}{{{\mathrm{IPCA}}2{\mathrm{SS}}}} \times {\mathrm{IPCA}}1\;{\mathrm{score}}} \right)}^2}+{{\left( {{\mathrm{IPCA}}2\;{\mathrm{score}}} \right)}^2}}$$

where IPCA1SS/IPCA2SS is the weight given to the IPCA1 value by dividing the IPCA1 sum of squares by the IPCA2 sum of squares. Lower ASV values were indicative of higher trait stability across the tested vinasse conditions, allowing for a precise and objective ranking of the parameters.

The GGE biplot analysis was employed to visually assess the genotype by trait (GT) interaction. It was used to identify the most discriminating and representative measured traits across different vinasse levels. By utilizing the ‘which-won-where’ polygon view, the study determined which single cross excelled in specific agronomic parameters. Furthermore, the vector scaling in the GGE biplot facilitated the identification of inter-relationships among measured traits^15,35^. Both AMMI and GGE biplot analyses were performed using GenStat^36^, followed by the method of Ye et al.^33^

## Results

### Analysis of variance

The results of the analysis of variance for physiological and yields components traits of three single cross s and fertilizer with different dose of vinasse are presented in Table 4. Significant differences were observed among vinasse doses, single cross, and their interactions for all studied traits across both seasons, except for the interaction between vinasse dose and single cross in oil percentage in both seasons as well as single cross s in 2023 season which were not significant (Table 4).

**Table 4:** Analysis of variance for the growth and physiology traits under vinasse fertilizer levels for each cross in the two seasons.

Variable	Source of variation	Vinasse fertilizer (VF)	Error (a)	Single cross (SC)	Error (b)	VF*SC	Error (c)
	DF	3	6	2	4	6	12
	Year	Mean square value					
Plant height cm	2022	1067.9**	0.58	135.3**	1.55	27.53**	0.77
	2023	887.8**	2.14	210.4**	2.47	10.74**	2.52
Number leaves/plant	2022	25.2**	0.12	18.31**	0.40	1.42**	0.05
	2023	24.2**	0.05	26.14**	0.05	1.01**	0.03
Flag leaf area	2022	5.59**	0.69	49.21**	2.18	6.99**	0.30
	2023	3.21**	0.20	66.65**	0.35	4.55**	0.18
Chlorophyll.	2022	47753.1**	16.7	16,531**	10.9	1848.6**	7.06
	2023	50583.0**	38.9	17613.7**	31.90	1443.1**	52.92
Stem diameter cm	2022	1.03**	0.01	0.21**	0.0003	0.099**	0.02
	2023	0.84**	0.03	0.19*	0.03	0.08*	0.03
Ear height cm	2022	740.8**	0.70	265.8**	0.23	14.36**	0.35
	2023	690.7**	0.94	245.9**	2.52	19.35**	1.81
Ear diameter cm	2022	2.30**	0.01	1.64**	0.01	0.09**	0.01
	2023	1.80**	0.002	3.24**	0.01	0.05**	0.002
Ear length cm	2022	12.55**	0.01	25.55**	0.12	0.28**	0.01
	2023	13.58**	0.00	28.93**	0.01	0.09**	0.01
Grains yield/ plant g	2022	445.6**	1.39	833.7**	0.95	70.63**	1.24
	2023	228.0**	1.90	815.7**	0.76	22.44**	1.00
100 grain weight g	2022	74.72**	0.68	120.6**	2.14	0.61	0.53
	2023	79.79**	0.08	69.89**	0.02	1.38**	0.102
Sheeling percentage%	2022	5.30**	0.07	0.30*	0.05	0.33**	0.05
	2023	10.77**	0.1	2.33**	0.004	0.23*	0.06
Grain yield (ton ha^−1^)	2022	1.86**	0.08	7.15**	0.07	0.06	0.03
	2023	2.21**	0.04	2.60**	0.01	0.08	0.04
Protein%	2022	3.25**	0.001	9.94**	0.001	0.01*	0.004
	2023	4.13**	0.0002	10.71**	0.002	0.14**	0.001
Oil%	2022	0.77**	0.01	0.04*	0.004	0.01	0.004
	2023	1.07**	0.02	0.02	0.01	0.01	0.01

*and **Significant at 5 and 1% probability levels, respectively.

#### Growth characters

Among the three maize Single cross s, single cross SC2036 exhibited significantly superior performance in number of leaves per plant, chlorophyll content, and stem diameter. Additionally, higher vinasse fertility (3 L/plot) was associated with significantly increased plant height, number of leaves, chlorophyll content, and stem diameter, in both seasons (Table 5). The interaction between Single cross type and vinasse fertilization level was statistically significant, with SC2036 combined with 3 L/plot of vinasse consistently producing the highest values for most growth traits i.e. number of leaves/plant, chlorophyll l and stem diameter in both seasons.

#### Grain yield and its attributes

Single cross 2036 gave significantly higher grain yields and its components i.e. ear diameter, ear length,100 grain weight and sheelling % followed by single cross 2031 than single cross 168 (Tables 6 α 7) for both seasons.

Furthermore, among the fertility levels, application of doses or levels of vinasse, the high doses of vinasse (3 L/plot) recorded significantly higher grain yield and its components i.e. ear diameter, ear length, 100 grain weight and shelling % (Table 6) for both seasons.

The interaction between single crosse and fertility levels of vinasse were significant for most of grain yield and its components, and the better interaction was single cross 2036 with fertility levels of vinasse with 3 L/ plot for both seasons.

#### Grain quality

Variation in grain quality (protein and oil percentage) were recorded among the three Single cross s and fertility levels of vinasse (Table 7). Single cross 2036 produced of protein and oil percentage. Also, fertility level of vinasse with 3 L/ plot recorded higher protein and oil percentage for each single cross. The single cross 2036 and fertility level of 3 L/ plot gave higher protein and oil percentage for both seasons.

### Means performance

#### Growth characteristics

The application of vinasse fertilizer significantly enhanced all measured growth and physiological parameters of maize across both 2022 and 2023. Plant height increased progressively with vinasse levels, reaching the maximum at 3 L/plot (250.4 and 253.0 cm for single cross SC2031 in 2022 and 2023, respectively seasons. Similarly, the number of leaves per plant was highest at 3 L/plot, peaking at 20.3 in single cross SC2036 for season 2023. Chlorophyll content also rose substantially with increased vinasse, with SC2036 showing the highest value (532.7) at 3 L/plot in 2023 (Table 5). Stem diameter followed the same trend, attaining the maximum (5.97 cm) in single cross SC2036 under 3 L/plot in 2023 season (Table 5). Interestingly, while flag leaf area did not show a consistent increase with vinasse, but the single cross SC168 under dose of 3 L/P recorded the largest values overall (up to 40.42 cm^2^), indicating genotype-specific responses (Table 5).

Overall, SC2036 responded most favorably to vinasse application, particularly at 3 L/plot, making it the best-performing single cross in terms of growth and physiological traits. Year-to-year consistency in results confirms the reliability of vinasse as a bio-fertilizer for improving maize performance.

**Table 5:** Mean of plant height cm, number of leaves/plants, flag leaf area, chlorophyll, and stem diameter of maize as affected by vinasse fertilizer, single cross, and their interaction in 2022 and 2023 seasons.

Variable	Plant height cm ± SE		Number of leaves/plants ± SE		Flag leaf area ± SE		Chlorophyll ± SE		Stem diameter ± SE	
	2022	2023	2022	2023	2022	2023	2022	2023	2022	2023
Vinasse fertilizer										
Control	220.4 ± 0.49_d_	227.5 ± 1.04_d_	13.87 ± 0.30_d_	15.03 ± 0.29_d_	38.43 ± 0.64_a_	36.10 ± 0.47_ab_	310.4 ± 4.2_d_	323.9 ± 5.0_d_	2.14 ± 0.03_d_	2.15 ± 0.01_c_
1 L/plot	233.7 ± 0.96_c_	242.9 ± 1.82_c_	14.99 ± 0.21_c_	15.92 ± 0.33_c_	38.01 ± 0.31_a_	36.41 ± 0.47_a_	385.4 ± 6.9_c_	396.4 ± 7.1_c_	2.48 ± 0.3_c_	2.50 ± 0.02_b_
2 L/plot	238.9 ± 1.80_b_	246.4 ± 1.26_b_	16.68 ± 0.48_b_	17.34 ± 0.55_b_	37.00 ± 0.93_b_	35.88 ± 1.11_b_	445.4 ± 14.8_b_	459.9 ± 15.4_b_	2.65 ± 0.02_b_	2.71 ± 0.03_a_
3 L/plot	246.2 ± 1.70_a_	250.0 ± 1.26_a_	17.60 ± 0.56_a_	18.77 ± 0.58_a_	36.79 ± 0.92_b_	35.02 ± 0.84_c_	475.5 ± 18.8_a_	494.1 ± 17.8_a_	2.95 ± 0.13_a_	2.85 ± 0.14_a_
Single cross										
SC2031	237.3 ± 3.2_a_	245.8 ± 2.7_a_	15.97 ± 0.58_b_	17.11 ± 0.52_b_	37.24 ± 0.60_b_	35.09 ± 0.34_b_	421.0 ± 22.9_b_	436.6 ± 23.0_b_	2.57 ± 0.10_b_	2.49 ± 0.09_b_
SC2036	236.2 ± 3.4_a_	241.9 ± 2.7_b_	16.92 ± 0.51_a_	18.04 ± 0.51_a_	35.71 ± 0.38_b_	33.97 ± 0.33_c_	429.8 ± 22.6_a_	444.6 ± 22.8_a_	2.68 ± 0.12_a_	2.70 ± 0.12_a_
SC168	231.0 ± 2.2_b_	237.4 ± 2.5_c_	14.47 ± 0.26_c_	15.15 ± 0.28_c_	39.72 ± 0.27_a_	38.49 ± 0.34_a_	361.6 ± 12.0_c_	374.6 ± 13.4_c_	2.41 ± 0.07_c_	2.47 ± 0.07_b_
Control										
SC2031	221.3 ± 0.88_g_	230.4 ± 1.52_g_	13.43 ± 0.28_g_	14.90 ± 0.10_hi_	39.66 ± 0.87_ab_	36.41 ± 0.26_d_	310.6 ± 4.9_i_	329.3 ± 3.7_g_	2.13 ± 0.006_e_	2.16 ± 0.006_de_
SC2036	220.0 ± 0.58_g_	228.0 ± 0.21_g_	14.97 ± 0.20_e_	16.07 ± 0.23_g_	36.20 ± 0.55_f_	34.45 ± 0.50_f_	321.9 ± 6.0_h_	336.7 ± 4.0_g_	2.15 ± 0.006_de_	2.17 ± 0.006_de_
SC168	219.9 ± 1.07_g_	224.0 ± 0.58_h_	13.20 ± 0.12_g_	14.13 ± 0.09_j_	39.42 ± 0.29_bc_	37.43 ± 0.24_c_	298.7 ± 4.5_j_	305.8 ± 3.4_h_	2.13 ± 0.11_e_	2.11 ± 0.006_e_
1 L/plot										
SC2031	237.1 ± 0.67_e_	249.8 ± 0.98_bc_	15.03 ± 0.15e	16.20 ± 0.15f_g_	38.14 ± 0.46_de_	35.73 ± 0.33_de_	394.4 ± 4.8_e_	404.4 ± 2.8_de_	2.47 ± 0.009_bc_	2.52 ± 0.009_bcd_
SC2036	232.7 ± 0.88_f_	240.6 ± 0.49_ef_	15.63 ± 0.20_d_	16.87 ± 0.19_e_	37.26 ± 0.54_e_	35.35 ± 0.34_e_	402.6 ± 1.6_d_	415.4 ± 3.2_cd_	2.56 ± 0.009_bc_	2.57 ± 0.006_bc_
SC168	231.4 ± 0.98_f_	238.1 ± 0.59_f_	14.30 ± 0.17_f_	14.70 ± 0.10_i_	38.64 ± 0.44_cd_	38.16 ± 0.23_bc_	359.2 ± 4.6_g_	369.3 ± 3.3_f_	2.42 ± 0.07_cd_	2.42 ± 0.009_cde_
2 L/plot										
SC2031	240.2 ± 0.79_d_	249.9 ± 0.67_bc_	17.07 ± 0.41_c_	17.87 ± 0.13_d_	36.04 ± 0.81_fg_	34.52 ± 0.32_f_	472.4 ± 3.0_c_	486.9 ± 7.2_b_	2.62 ± 0.006_bc_	2.73 ± 0.009_bc_
SC2036	243.9 ± 2.00c	247.2 ± 0.56_cd_	18.03 ± 0.15_b_	18.93 ± 0.18_c_	34.56 ± 0.35_h_	32.98 ± 0.29_g_	476.9 ± 2.2_c_	493.4 ± 4.8_b_	2.73 ± 0.009_b_	2.79 ± 0.009_b_
SC168	232.7 ± 1.20_f_	242.1 ± 1.62_e_	14.93 ± 0.18_e_	15.23 ± 0.12_h_	40.41 ± 0.39_a_	40.14 ± 0.28a	386.8 ± 4.3_f_	399.4 ± 1.6_e_	2.59 ± 0.05_bc_	2.62 ± 0.009_bc_
3 L/plot										
SC2031	250.4 ± 0.98_a_	253.0 ± 1.07_a_	18.33 ± 0.20_b_	19.47 ± 0.24_b_	35.11 ± 0.32_gh_	33.69 ± 0.31_g_	506.7 ± 7.6_b_	525.8 ± 4.6_a_	3.07 ± 0.04_a_	2.56 ± 0.32_bc_
SC2036	248.3 ± 1.71_b_	251.8 ± 0.35_ab_	19.03 ± 0.15_a_	20.30 ± 0.15_a_	34.83 ± 0.35_h_	33.10 ± 0.26_g_	518.0 ± 5.8_a_	532.7 ± 7.8_a_	3.27 ± 0.04_a_	3.25 ± 0.006_a_
SC168	240.0 ± 0.58_d_	245.3 ± 0.88_d_	15.43 ± 0.09_d_	16.53 ± 0.29_f_	40.42 ± 0.0.20_a_	38.25 ± 0.64_b_	401.7 ± 4.7_d_	423.8 ± 4.3_c_	2.50 ± 0.18_bc_	2.73 ± 0.02_bc_

#### Grain yield and yield attributes

Vinasse fertilizer significantly influenced ear traits and grain yield in both 2022 and 2023. Increasing vinasse levels consistently reduced ear height, with the highest at control (120.6 cm) and the lowest at 3 L/plot (99.4 cm) in 2022. In contrast, ear diameter, ear length, grain yield plant^−1,^ and 100-grain weight, all improved as vinasse levels increased. The best overall performance was recorded at 3 L/plot, with SC2036 single cross producing the largest ear diameter (5.20–5.97 cm), ear length (19.70, 20.73 cm), grain yield (187.0, 191.2 g/plant), and 100-grain weight (35.17,38.43 g) in 2022 and 2023 respectively (Table 6).

Among the single cross s, SC2036 outperformed SC2031 and SC168 in most traits, particularly under 3 L/plot treatment. While single cross SC168, producing the tallest ears under control, consistently had smaller ear diameter, ear length, and yield across all vinasse levels. Interaction effects further confirmed that the highest yield and grain quality were achieved when SC2036 was combined with 3 L/plot vinasse, whereas SC168 under control had the lowest values for nearly all traits.

Overall, the results show that higher vinasse application (3 L/plot) improves maize productivity significantly, especially when paired with the SC2036 single cross, suggesting a strong positive interaction between vinasse fertilization and single cross genotype for yield-related traits.

**Table 6:** Mean of ear height, ear diameter cm, ear length cm, grain yield/plant g, and 100-grain weight g of maize as affected by vinasse fertilizer, single cross, and interaction in 2022 and 2023 seasons.

Variable	Ear height cm ±		Ear diameter cm ±		Ear length cm ±		Grains yield/ plant g ±		100-grain weight g ±	
	2022	2023	2022	2023	2022	2023	2022	2023	2022	2023
Vinasse fertilizer										
Control	120.6 ± 0.86_a_	117.0 ± 0.87_a_	3.71 ± 0.07_d_	4.45 ± 0.12_d_	16.07 ± 0.48_d_	16.96 ± 0.48_d_	162.4 ± 0.45_d_	173.3 ± 2.0_d_	26.36 ± 1.07_d_	29.43 ± 0.73_d_
1 L/plot	113.4 ± 1.47_b_	109.5 ± 1.27_b_	4.44 ± 0.17_c_	4.78 ± 0.13_c_	17.09 ± 0.51_c_	17.98 ± 0.46_c_	173.2 ± 2.87_c_	179.5 ± 2.7_c_	28.32 ± 0.86_c_	31.57 ± 0.67_c_
2 L/plot	106.7 ± 2.21_c_	103.4 ± 2.28_c_	4.66 ± 0.11_b_	5.17 ± 0.17_b_	17.86 ± 0.37_b_	19.06 ± 0.47_b_	175.2 ± 2.98_b_	181.7 ± 2.7_b_	30.79 ± 0.89_b_	34.02 ± 0.58_b_
3 L/plot	99.4 ± 1.03_d_	96.4 ± 1.21_d_	4.87 ± 0.10_a_	5.47 ± 0.19_a_	18.86 ± 0.35_a_	19.76 ± 0.41_a_	178.7 ± 3.54_a_	185.2 ± 2.6_a_	32.97 ± 1.00_a_	36.30 ± 0.91_a_
Single cross										
SC2031	109.4 ± 2.4_b_	105.3 ± 2.5_b_	4.55 ± 0.13_b_	5.09 ± 0.14_b_	17.92 ± 0.31_b_	18.95 ± 0.32_b_	176.7 ± 2.6_a_	183.0 ± 2.0_b_	30.79 ± 0.1_a_	33.47 ± 0.93_b_
SC2036	105.6 ± 2.6_c_	102.8 ± 2.4_c_	4.71 ± 0.15_a_	5.41 ± 0.14_a_	18.64 ± 0.25_a_	19.67 ± 0.30_a_	177.7 ± 2.6_a_	186.2 ± 1.2_a_	32.02 ± 0.76_a_	34.86 ± 0.75_a_
SC168	114.9 ± 2.3_a_	111.6 ± 2.2_a_	4.00 ± 0.13_c_	4.40 ± 0.09_c_	15.83 ± 0.38_c_	16.69 ± 0.35_c_	162.8 ± 0.54_b_	170.6 ± 1.0_c_	26.02 ± 0.81_b_	30.16 ± 0.70_c_
Interaction										
Control										
SC2031	119.9 ± 0.27_b_	115.6 ± 0.32_b_	3.80 ± 0.01_f_	4.51 ± 0.01_g_	16.57 ± 0.09_g_	17.50 ± 0.17_g_	162.4 ± 0.50_ghi_	171.8 ± 0.83_f_	27.27 ± 0.07_a_	29.72 ± 0.31_h_
SC2036	118.2 ± 0.32_c_	115.0 ± 0.49_b_	3.87 ± 0.01_f_	4.83 ± 0.02_e_	17.40 ± 0.06_f_	18.27 ± 0.09_f_	163.9 ± 0.26_fg_	180.8 ± 0.53_d_	29.50 ± 0.26_a_	31.73 ± 0.27_g_
SC168	123.7 ± 0.87_a_	120.3 ± 0.33_a_	3.45 ± 0.04_g_	4.01 ± 0.01_i_	14.23 ± 0.12_j_	15.10 ± 0.06_j_	160.9 ± 0.07_i_	167.2 ± 1.01_g_	22.30 ± 0.12_a_	26.85 ± 0.27_i_
1 L/plot										
SC2031	113.5 ± 0.64_e_	110.3 ± 0.15_d_	4.73 ± 0.01_c_	4.82 ± 0.01_e_	17.47 ± 0.09_f_	18.53 ± 0.12_e_	178.5 ± 1.17_e_	184.8 ± 0.43_c_	29.40 ± 0.10_a_	31.61 ± 0.21_g_
SC2036	108.3 ± 0.64_f_	104.8 ± 0.31_e_	4.83 ± 0.02_bc_	5.20 ± 0.12_d_	18.63 ± 0.03_d_	19.20 ± 0.06_d_	179.2 ± 0.50_de_	184.6 ± 0.54_c_	30.60 ± 0.20_a_	33.80 ± 0.35e
SC168	118.3 ± 0.40_c_	113.4 ± 0.35_bc_	3.75 ± 0.02_f_	4.31 ± 0.01_h_	15.17 ± 0.09_i_	16.20 ± 0.12_i_	161.8 ± 0.33_hi_	168.9 ± 1.50_g_	24.97 ± 0.26_a_	29.30 ± 0.36_h_
2 L/plot										
SC2031	105.2 ± 0.64_g_	100.3 ± 0.33_f_	4.78 ± 0.01_bc_	5.33 ± 0.02_c_	18.30 ± 0.06_e_	19.40 ± 0.12_c_	181.5 ± 1.00_c_	185.7 ± 0.84_c_	32.23 ± 0.22_a_	34.84 ± 0.09_d_
SC2036	99.9 ± 0.20_i_	97.5 ± 0.25_g_	4.92 ± 0.02_b_	5.65 ± 0.08_b_	18.83 ± 0.03_c_	20.47 ± 0.09_b_	180.6 ± 0.27_cd_	188.3 ± 0.82_b_	32.80 ± 0.70_a_	35.47 ± 0.19_c_
SC168	114.9 ± 0.22_d_	112.3 ± 0.33_cd_	4.29 ± 0.17_e_	4.53 ± 0.02_g_	16.43 ± 0.22_h_	17.30 ± 0.17_h_	163.5 ± 1.27_fgh_	171.3 ± 1.32_f_	27.33 ± 0.15_a_	31.77 ± 0.23_g_
3 L/plot										
SC2031	99.1 ± 0.49_i_	94.8 ± 2.35_h_	4.88 ± 0.01_b_	5.71 ± 0.01_b_	19.37 ± 0.09_b_	20.37 ± 0.15_b_	184.3 ± 1.35_b_	189.5 ± 0.25_ab_	34.27 ± 0.28_a_	37.73 ± 0.27_b_
SC2036	96.1 ± 0.35_j_	94.1 ± 0.58_h_	5.20 ± 0.12_a_	5.97 ± 0.04_a_	19.70 ± 0.12_a_	20.73 ± 0.09_a_	187.0 ± 1.52_a_	191.2 ± 1.18_a_	35.17 ± 1.59_a_	38.43 ± 0.17_a_
SC168	103.0 ± 0.55_h_	100.3 ± 0.33_f_	4.52 ± 0.02_d_	4.73 ± 0.02_f_	17.50 ± 0.29_f_	18.17 ± 0.12_f_	164.9 ± 0.35_f_	175.0 ± 1.00_e_	29.47 ± 0.07_a_	32.73 ± 0.32_f_

Means followed by the same letter are not significant *p* < 0.05 level according to the least significantly different multiple comparison test. ±SE= Standard error.

#### Grain quality

The application of vinasse fertilizer had a significant impact on shelling percentage, grain yield, and grain quality traits (protein and oil content) in both growing seasons. Notably, shelling percentage slightly declined as vinasse levels increased, from 81.39% under control to 78.86% at 3 L/plot in 2023 season. However, the grain yield showed a steady increase, reaching a maximum of 8.07 t/ha (2023) under 3 L/plot, compared to only 6.87 t/ha under control. Similarly, protein content improved significantly with higher vinasse levels, peaking at 13.23% in 2023, while oil content also rose, achieving 5.63% at 3 L/plot (Table 7).

Among the single cross s, SC2036 under 3 L//p vinasse exhibited the best performance in both years, with the highest grain yield (8.43, 8.60 t/ha), protein content (13.89,14.14%), and oil percentage (5.55, 5.65%) in 2022 and 2023, seasons. In contrast, SC168 had the lowest grain yield and protein content, though its oil content remained competitive. Interaction data confirmed that the combination of SC2036 with 3 L/plot vinasse consistently produced the highest values across all traits, including the top protein (14.14%) and oil (5.65%) contents in 2023 season (Table 7). These results clearly suggest that vinasse application at 3 L/plot, particularly when combined with SC2036, greatly enhances maize productivity and grain nutritional quality, making it the most effective treatment across both years.

**Table 7:** Mean of sheelling percentage, grain yield (ton/ha^−1^) protein %, and oil % of maize as affected by vinasse fertilizer, single cross, and their interaction in 2022 and 2023 seasons.

Variable	Sheelling percentage % ±		Grain yield (ton/ha^−1^) ±		Protein % ±		Oil % ±	
	2022	2023	2022	2023	2022	2023	2022	2023
Vinasse fertilizer								
Control	80.85 ± 0.14_a_	81.39 ± 0.16_a_	6.53 ± 0.21_c_	6.87 ± 0.12_c_	11.55 ± 0.26_d_	11.76 ± 0.27_d_	4.83 ± 0.02_c_	4.90 ± 0.01_c_
1 L/plot	80.22 ± 0.12_b_	79.73 ± 0.16_b_	6.96 ± 0.26_b_	7.33 ± 0.20_b_	11.86 ± 0.28_c_	12.16 ± 0.36_c_	4.91 ± 0.02_c_	4.98 ± 0.02_c_
2 L/plot	79.60 ± 0.14_c_	79.36 ± 0.21_c_	7.28 ± 0.26_ab_	7.51 ± 0.14_b_	12.54 ± 0.26_b_	12.93 ± 0.23_b_	5.25 ± 0.03_b_	5.37 ± 0.04_b_
3 L/plot	79.08 ± 0.10_d_	78.86 ± 0.13_d_	7.60 ± 0.28_a_	8.07 ± 0.14_a_	12.85 ± 0.26_a_	13.23 ± 0.23_a_	5.46 ± 0.06_a_	5.63 ± 0.06_a_
Single cross								
SC2031	80.07 ± 0.22_a_	79.88 ± 0.31_a_	7.17 ± 0.16_b_	7.24 ± 0.11_b_	11.59 ± 0.17_c_	11.89 ± 0.20_c_	5.11 ± 0.9a_b_	5.18 ± 0.09_a_
SC2036	79.76 ± 0.15_b_	80.26 ± 0.24_b_	7.82 ± 0.20_a_	7.98 ± 0.16_a_	13.25 ± 0.16_a_	13.60 ± 0.15_a_	5.17 ± 0.09_a_	5.25 ± 0.09_a_
SC168	79.97 ± 0.28_ab_	79.38 ± 0.34_c_	6.28 ± 0.11_c_	7.12 ± 0.15_b_	11.76 ± 0.15_b_	12.06 ± 0.20_b_	5.05 ± 0.07_b_	5.23 ± 0.10_a_
Interaction								
Control								
SC2031	80.80 ± 0.10_b_	81.44 ± 0.23_a_	6.66 ± 0.28_a_	6.81 ± 0.09_a_	10.87 ± 0.03_j_	11.11 ± 0.05_k_	4.84 ± 0.02_a_	4.88 ± 0.01_a_
SC2036	80.46 ± 0.07_bc_	81.56 ± 0.12_a_	7.04 ± 0.31_a_	7.28 ± 0.06_a_	12.55 ± 0.03_d_	12.84 ± 0.04_d_	4.88 ± 0.04_a_	4.94 ± 0.02_a_
SC168	81.28 ± 0.21_a_	81.16 ± 0.42_a_	5.89 ± 0.16_a_	6.52 ± 0.01_a_	11.22 ± 0.01_i_	11.33 ± 0.04_j_	4.77 ± 0.03_a_	4.87 ± 0.003_a_
1 L/plot								
SC2031	80.52 ± 0.22_bc_	80.32 ± 0.0_b_	7.00 ± 0.26_c_	7.07 ± 0.03_a_	11.25 ± 0.06_i_	11.41 ± 0.06_i_	4.89 ± 0.01_a_	4.94 ± 0.01_a_
SC2036	79.90 ± 0.10_d_	79.58 ± 0.10_cd_	7.73 ± 0.32_a_	8.10 ± 0.06_a_	12.95 ± 0.03_c_	13.59 ± 0.06_c_	4.93 ± 0.03_a_	5.02 ± 0.05_a_
SC168	80.25 ± 0.03_cd_	79.29 ± 0.15_d_	6.14 ± 0.11_a_	6.83 ± 0.09_a_	11.37 ± 0.01_h_	11.47 ± 0.03_h_	4.90 ± 0.05_a_	4.97 ± 0.03_a_
2 L/plot								
SC2031	79.97 ± 0.17_d_	79.96 ± 0.03_bc_	7.37 ± 0.19_a_	7.30 ± 0.06_a_	11.95 ± 0.03_g_	12.35 ± 0.08_g_	5.20 ± 0.02_a_	5.27 ± 0.03_a_
SC2036	79.34 ± 0.18_ef_	79.55 ± 0.12_d_	8.07 ± 0.26_a_	7.93 ± 0.32_a_	13.58 ± 0.04_b_	13.85 ± 0.04_b_	5.32 ± 0.05_a_	5.40 ± 0.04_a_
SC168	79.48 ± 0.24_e_	78.58 ± 0.03_e_	6.40 ± 0.15_a_	7.30 ± 0.06_a_	12.09 ± 0.05_f_	12.58 ± 0.04_f_	5.22 ± 0.05_a_	5.44 ± 0.09_a_
3 L/plot								
SC2031	79.00 ± 0.15_fg_	79.31 ± 0.10_d_	7.67 ± 0.24_a_	7.78 ± 0.04_a_	12.29 ± 0.02_e_	12.69 ± 0.05_e_	5.50 ± 0.15_a_	5.63 ± 0.17_a_
SC2036	79.35 ± 0.20_ef_	78.80 ± 0.03_e_	8.43 ± 0.34_a_	8.60 ± 0.06_a_	13.89 ± 0.02_a_	14.14 ± 0.07_a_	5.55 ± 0.05_a_	5.65 ± 0.06_a_
SC168	78.88 ± 0.03_g_	78.48 ± 0.13_e_	6.69 ± 0.11_a_	7.82 ± 0.02_a_	12.37 ± 0.02_e_	12.85 ± 0.04_d_	5.31 ± 0.07a	5.62 ± 0.13_a_

Means followed by the same letter are not significant *p* < 0.05 level according to the least significantly different multiple comparison test. ±SE= Standard error.

### Correlation analysis on traits in three single crosse

A positive correlation was observed between leaf number (NL) and several traits, including plant height (PH), flag leaf area (ALF), chlorophyll content (Chl), stem diameter (SD), ear diameter (ED), ear length (EL), grain yield (GYP), 100-grain weight (PP), and protein (OP) content for all three single-cross Single cross s in both seasons. For SC2031, correlations ranged from 0.91 to 0.99, while negative correlations were found with ear height (EH, -1.00) and shelling percentage (SP, − 0.96) (Table S1). SC2036 showed similar positive correlations with NL, ranging from 0.87 to 0.99, and negative correlations with EH (− 0.95) and SP (− 0.92). SC168 exhibited positive correlations from 0.85 to 0.99, and negative correlations with EH (− 0.93) and SP (− 0.99). Chlorophyll content (Chl) also exhibited strong positive correlations with SD, ED, EL, GYP, PP, and OP. In SC2031, these ranged from 0.90 to 0.99, with negative correlations with EH (− 0.97) and SP (− 0.98). SC2036 showed positive correlations from 0.91 to 0.97, and negative correlations with EH (− 0.94) and SP (− 0.88). SC168 exhibited positive correlations from 0.86 to 0.96, and negative correlations with EH (− 0.87) and SP (− 0.98). Ear height (EH) was negatively correlated with PH, NL, ALF, Chl, SD, ED, EL, GYP, PP, and OP. In SC2031, correlations ranged from − 0.86 to − 1.00; SC2036 ranged from − 0.87 to − 0.99, and SC168 ranged from − 0.67 to − 0.96. Shelling percentage (SP) showed strong negative correlations with PH, NL, ALF, Chl, SD, EL, GYP, PP, and OP, but positive correlations with EH for all single cross s. For SC2031, correlations ranged from − 0.78 to − 0.96 (negative) and 0.98 (positive with EH); SC2036 ranged from − 0.79 to − 0.93 (negative) and 0.87 (positive); SC168 ranged from − 0.84 to − 0.99 (negative) and 0.81 (positive with EH) (Table S1).

### Trait evaluation using AMMI model in maize single cross

 AMMI (Additive Main Effects and Multiplicative Interaction) analysis of variance for maize Single crosses SC2031, SC2036, and SC168 under four vinasse treatments across the 2022 and 2023 seasons are shown in (Tables S2, S3, and S4). In both seasons, the analysis for the three crosses revealed that most of the total sum of squares (SS) is attributed to the traits (over 96% of the total variation). This indicates that the inherent differences between the measured traits are the primary source of variation. The treatments (Vinasse levels) and the interactions are also highly significant (*P* < 0.01), confirming that vinasse applications significantly altered trait expression.

Principal component analysis of the interaction revealed that IPCA1 captured most of the interaction variance—99.72% in 2022 and 99.54% in 2023—highlighting a dominant interaction pattern. IPCA2 explained only a small fraction of the variance (0.20% in 2022 and 0.44% in 2023). Minimal error and residual SS confirmed the robustness and reliability of the AMMI model.

For single cross SC2036, the AMMI analysis under the same vinasse treatments showed significant variation across traits and environments in both years. The total SS was 2,420,079 in 2022 and 2,577,011 in 2023. Vinasse treatments accounted for the largest portion of this variation (SS = 2,419,516 in 2022 and 2,576,320 in 2023), demonstrating their strong influence on growth and yield traits. Trait contributions were comparatively smaller (SS = 7065 in 2022 and 6239 in 2023), suggesting that environmental factors, particularly vinasse application, played a more dominant role.

The interaction between vinasse treatments and traits (GEI) was also significant, with SS values of 63,359 (2022) and 63,709 (2023). IPCA1 explained over 99% of this interaction in both years, indicating that the primary interaction pattern was effectively captured. IPCA2 and residuals contributed minimally, confirming a good model fit and low unexplained variation.

For single cross SC168, the AMMI analysis under four vinasse treatments revealed significant variation in trait performance across both years, with total SS values of 1,826,511 (2022) and 1,950,223 (2023). Treatment effects accounted for nearly all the variation (99.98% in both years), as reflected by SS values of 1,825,974 (2022) and 1,949,891 (2023). Trait contributions were also substantial, representing approximately 98.8% (2022) and 98.7% (2023) of the total variation.

The interaction between vinasse treatments and traits contributed a smaller but notable portion of the variation (1.0% in 2022 and 1.15% in 2023), indicating differential genotype responses to vinasse levels. IPCA1 explained most of the GEI variance (98.84% in 2022 and 99.4% in 2023), effectively capturing genotype stability and adaptability. IPCA2 contributed minimally (1.03% in 2022 and 0.58% in 2023), and residuals were negligible. Error variance was low in both years, confirming high experimental precision.

#### AMMI biplot results of single cross maize SC2031 under different vinasse fertilizer treatments across two seasons (2022 and 2023)

**Fig. 1:**
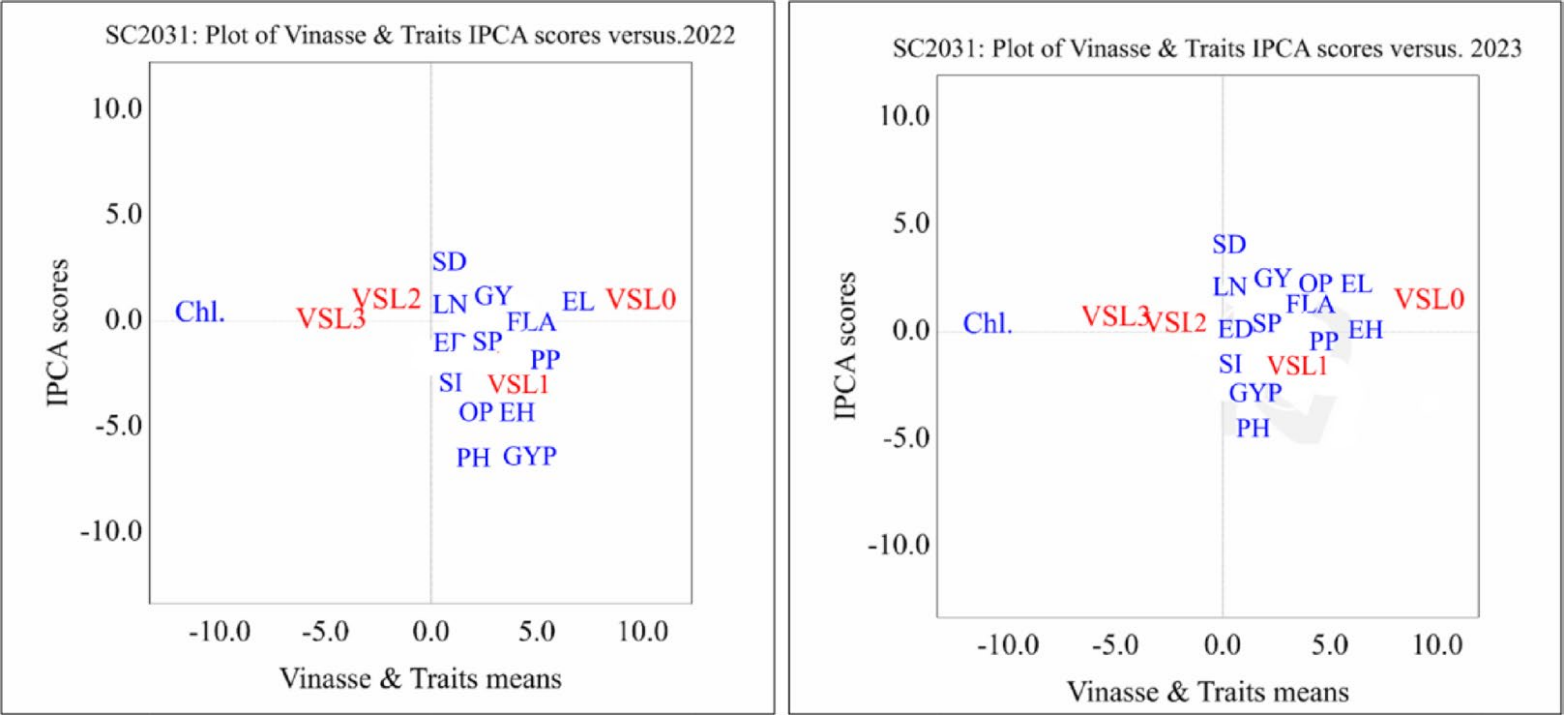
Biplot average traits and Vinasse of single cross SC 2031 on different treatment levels at principal component values (AMMI). PH, plant height; LN, leaf number; FLA, Falge leaf area; Chl., chlorophyll; SD, stem diameter; EH, ear height; ED, ear diameter; EL, ear length; GYP, grains yield/plant; SI, 100-grain weight; SP, sheeling percentage; GY, grain yield; PP, protin percentage; OP, oil percentage. 0 until 4 Vinasse.

The AMMI (Additive Main Effects and Multiplicative Interaction) biplots for the single cross SC2031 across the 2022 and 2023 growing seasons revealed clear interactions between vinasse fertilizer levels and key agronomic traits. In both seasons, vinasse level 0 (VSL0 – control) was distinctly separated from most traits, indicating limited contribution to trait enhancement. Conversely, vinasse levels 2 and 3 (VSL2 and VSL3) were closely associated with important yield components such as ear height (EH), ear diameter (ED), stem diameter (SD), grain yield per plant (GYP), and 100-grain weight (SI), particularly in the 2023 season, signifying a positive impact of higher vinasse doses on productivity in Fig. 1.

In the 2022 season, the AMMI biplot analysis revealed notable interactions between vinasse fertilizer levels and key agronomic traits of Single cross SC2031. Vinasse level 0 (VSL0), the control, was located far on the positive side of the x-axis, indicating minimal association with most traits. Traits such as plant height (PH), ear height (EH), stem diameter (SD), and ear diameter (ED) clustered near the origin, suggesting stable expression across treatments. Grain yield per plant (GYP), protein percentage (PP), and 100-grain weight (SI) were positioned moderately near VSL1 and VSL2, implying that these lower to moderate vinasse applications contributed positively to yield-related traits. In contrast, chlorophyll content (Chl.) appeared isolated on the negative side of the biplot, indicating a weaker or negative association with vinasse levels, particularly higher ones.

In 2023, traits like plant height (PH), grain yield (GY), and protein percentage (PP) also aligned more closely with VSL2 and VSL3, indicating improved nutritional and agronomic performance at these fertilization levels. Chlorophyll content (Chl.), however, was consistently positioned on the negative side of the biplot in both years, isolated from other traits and vinasse levels, suggesting a divergent response or reduced sensitivity to vinasse application. The trait vectors were more tightly clustered in 2023, implying stronger and more uniform trait expressions under vinasse treatments that year.

Overall, the biplots suggest that higher vinasse application levels, particularly VSL2 and VSL3, enhance the performance of SC2031 by positively influencing yield-related traits and nutritional quality, whereas lower vinasse levels or the control condition were less effective.

#### AMMI biplot results of Single cross maize SC2036 under different vinasse fertilizer treatments across two seasons (2022 and 2023)

**Fig. 2:**
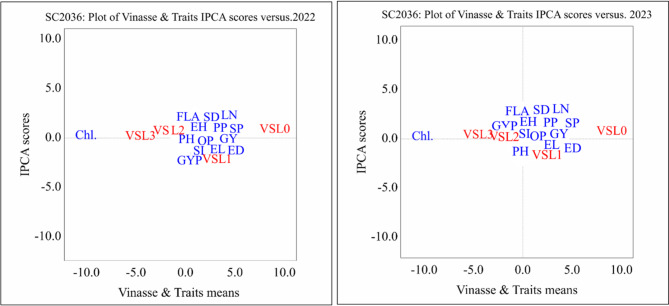
Biplot average traits and Vinasse of single cross SC 2036 on different treatment levels at principal component values (AMMI). PH, plant height; LN, leaf number; FLA, Falge leaf area; Chl., chlorophyll; SD, stem diameter; EH, ear height; ED, ear diameter; EL, ear length; GYP, grains yield/plant; SI, 100-grain weight; SP, sheeling percentage; GY, grain yield; PP, protin percentage; OP, oil percentage. 0 until 4 Vinasse.

The AMMI biplot results showed that the control treatment (VSL0) was consistently separated from most traits, indicating its limited effect on improving growth and yield of SC2036. Most agronomic traits were located near the biplot origin, suggesting relatively stable expression across vinasse levels, while chlorophyll content showed a weak and inconsistent response in Fig. 2.

Moderate to high vinasse levels (VSL2 and VSL3) were more closely associated with key yield and growth traits, particularly in the 2023 season, reflecting a more favorable response under these treatments. General, the results indicate that SC2036 responds better to moderate–high vinasse application, whereas the control and low levels provide limited benefits.

#### AMMI biplot results of Single cross maize SC168 under different vinasse fertilizer treatments across two seasons (2022 and 2023)

**Fig. 3:**
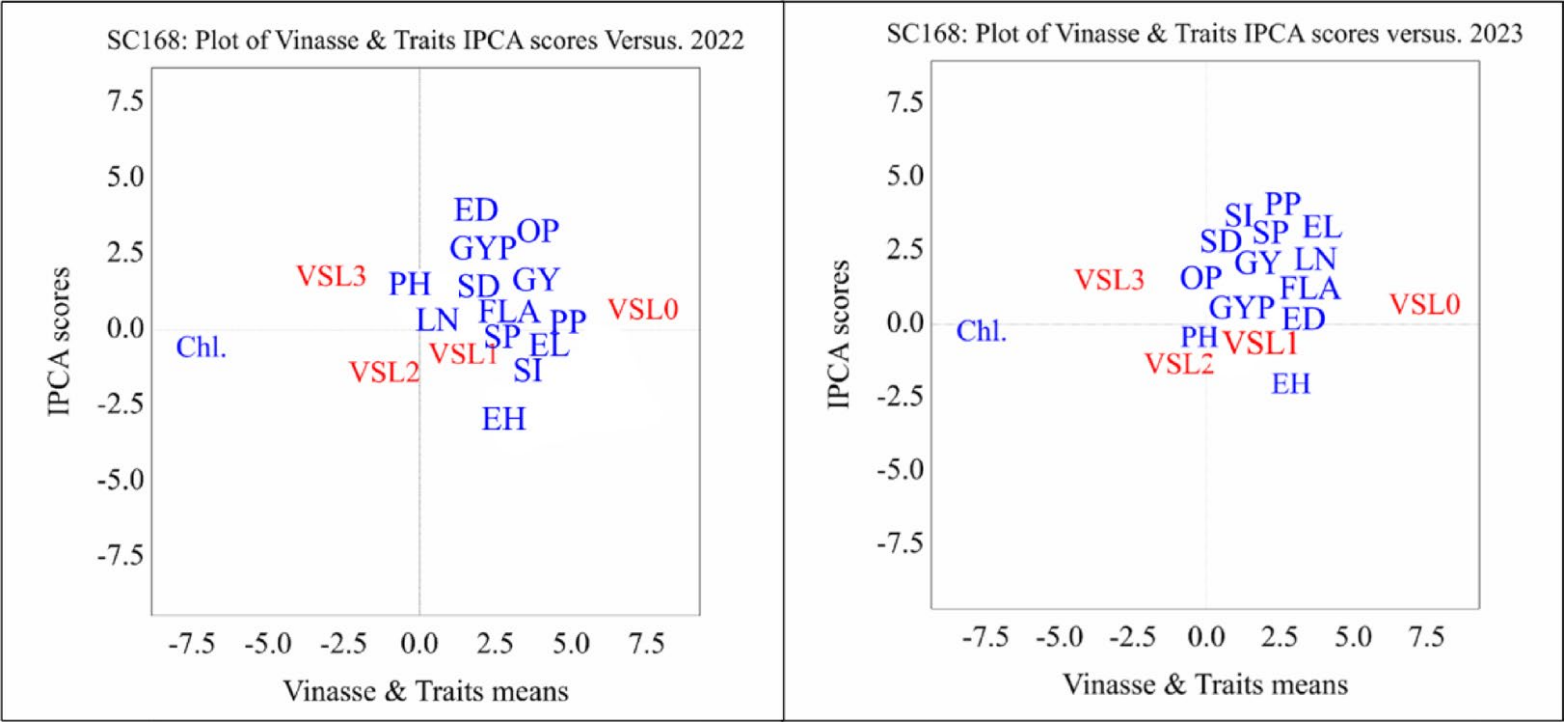
Biplot average traits and Vinasse of single cross SC168 on different treatment levels at principal component values (AMMI). PH, plant height; LN, leaf number; FLA, Falge leaf area; Chl., chlorophyll; SD, stem diameter; EH, ear height; ED, ear diameter; EL, ear length; GYP, grains yield/plant; SI, 100-grain weight; SP, sheeling percentage; GY, grain yield; PP, protin percentage; OP, oil percentage. 0 until 4 Vinasse.

The AMMI biplot analysis for the single cross SC168 in the 2022 season showed a clear separation of the control treatment (VSL0) from most agronomic traits, indicating its limited contribution to improving growth and yield performance. Moderate to higher vinasse levels, particularly VSL1 and VSL3, were more closely associated with vegetative growth traits such as plant height, stem diameter, and flag leaf area, suggesting a beneficial role of vinasse in promoting plant growth. In contrast, ear height and chlorophyll content exhibited divergent positions on the biplot, reflecting weak or inconsistent responses to vinasse application during this season in Fig. 3.

In the 2023 season, the association between moderate vinasse levels (VSL1 and VSL2) and key yield traits, especially grain yield per plant, became more pronounced, indicating an improved response of SC168 under these treatments. The continued separation of the control treatment confirms its limited effectiveness, while the consistent isolation of chlorophyll content across both seasons suggests that this trait is less influenced by vinasse fertilization. Overall, these results indicate that SC168 benefits mainly from moderate vinasse application, showing enhanced growth and yield stability compared with the control.

### Trait stability using AMMI stability value (ASV)

Table 8 and Table S5 summarize the AMMI Stability Value (ASV) for 14 different traits across the four vinasse levels for the three maize single cross s (SC2031, SC2036, and SC168) during the 2022 and 2023 growing seasons. It could be observed that the single cross **SC2031** recorded the lowest ASV values across all measured traits in both years. For instance, in 2022, traits like SI (16.0), GYP (42.1), and EL (47.0) showed remarkable stability. This suggests that SC2031 is highly resilient, maintaining consistent trait expression regardless of environmental fluctuations or treatment changes. In contrast, the cross **SC2036** exhibited the highest ASV scores, particularly for Chlorophyll content (Chl.) which reached 22,097.1 in 2023. High ASV values indicate that these traits in SC2036 are extremely sensitive to environmental conditions, showing high variability rather than stability.

**Table 8:** AMMI stability values (ASV) for each single maize cross across vinasse levels in both seasons.

Trait means and scores	ASV					
	SC2031		SC2036		SC168	
	2022	2023	2022	2023	2022	2023
FLA	63.3	120.5	826.0	2177.9	692.7	270.6
Chl.	782.5	1481.0	7795.7	22097.1	5797.7	2582.9
ED	63.8	131.9	686.0	1809.2	511.6	215.1
EH	217.4	382.0	1707.2	4497.6	1202.5	531.6
EL	47.0	100.0	649.4	1631.5	459.8	193.1
GY	66.4	124.5	681.6	1815.3	512.8	219.4
GYP	42.1	42.0	179.5	702.2	129.6	5.8
NL	54.5	111.8	554.8	1427.8	388.1	169.1
OP	68.1	131.1	709.4	1860.7	524.0	221.6
PH	80.1	154.9	507.5	889.2	300.0	57.6
PP	63.2	119.9	680.1	1796.4	498.0	208.3
SD	69.1	133.1	695.8	1826.8	517.5	225.6
SI	16.0	58.7	501.4	1191.1	323.1	120.6
SP	91.8	180.4	791.1	2249.8	597.1	259.8

FLA, Falge leaf area; Chl., chlorophyll; ED, ear diameter; EH, ear height; EL, ear length; GY, grain yield/ plot; GYP, grains yield plant-1; NL, number of leaves/plant; OP, oil percentage; PH, plant height; protin percentage; PP, stem diameter; SI, 100-grain weight; SP, sheeling percentage.

### The significance of the difference between the stability of different traits

The grain yield (GY) and grain yield per plant (GYP) for SC2031 showed much lower ASV values compared to the other single cross s, implying that its yield is more reliable across different vinasse conditions. For morphological traits such as plant height (PH) and number of leaves (NL) followed the same pattern, with SC2031 being the most stable and SC2036 being the most responsive/unstable. In the case of SC168, this single cross occupied an intermediate position. Interestingly, in 2023, its stability improved significantly for several traits (e.g., GYP dropped to 5.8), suggesting a better adaptation to the specific conditions of the second season compared to the first.

Table 9 and Table S6 show the AMMI Stability Value (ASV) for three single cross s (SC2031, SC2036, and SC168) across four vinasse levels (VSL) during the 2022 and 2023 seasons. Based on the ASV scores, SC2031 consistently exhibits the lowest values across all treatment levels in both years. In 2022, its ASV ranged from 30.2 to 667.3, which is lower than the scores for SC2036 and SC168. This indicates that SC2031 is the most stable single cross in this study, as lower ASV values signify a more consistent performance and less sensitivity to changes in vinasse application. Furthermore, SC2036 recorded the highest ASV values, reaching a peak of 16,727.4 at VSL0 in 2023. Such high magnitude in ASV suggests that this single cross is highly responsive and potentially unstable across different conditions. While it might show high yield potential, its performance fluctuates drastically depending on the vinasse level. The VSL1 treatment appears to be the most “stable” environment for all single cross s, as evidenced by the lowest ASV scores recorded for that level (e.g., 30.2 for SC2031 and 579.6 for SC168 in 2022). Conversely, the control or initial levels (VSL0) generally showed higher ASV scores, indicating greater interaction complexity at those points.

**Table 9:** AMMI stability values (ASV) for each cross-trait level.

Vanasse means and scores	ASV					
	SC2031		SC2036		SC168	
	2022	2023	2022	2023	2022	2023
VSL0	667.3	1233.2	5984.7	16727.4	4484.5	1923.9
VSL1	30.2	92.4	1504.0	4463.2	1081.9	579.6
VSL2	254.1	425.8	2619.1	7589.7	2072.5	901.8
VSL3	443.4	899.8	4869.6	13600.9	3493.9	1601.8

Vinasse solution plus: The applied rates of a control 0 L/plot (VSL0), 1 L/plot (VSL1), 2 L/plot (VSL2), and 3 L/plot (VSL3) (10.5 m^2^).

#### GGE biplot analysis of vinasse levels and traits for single cross SC2031 (2022 and 2023)

**Fig. 4:**
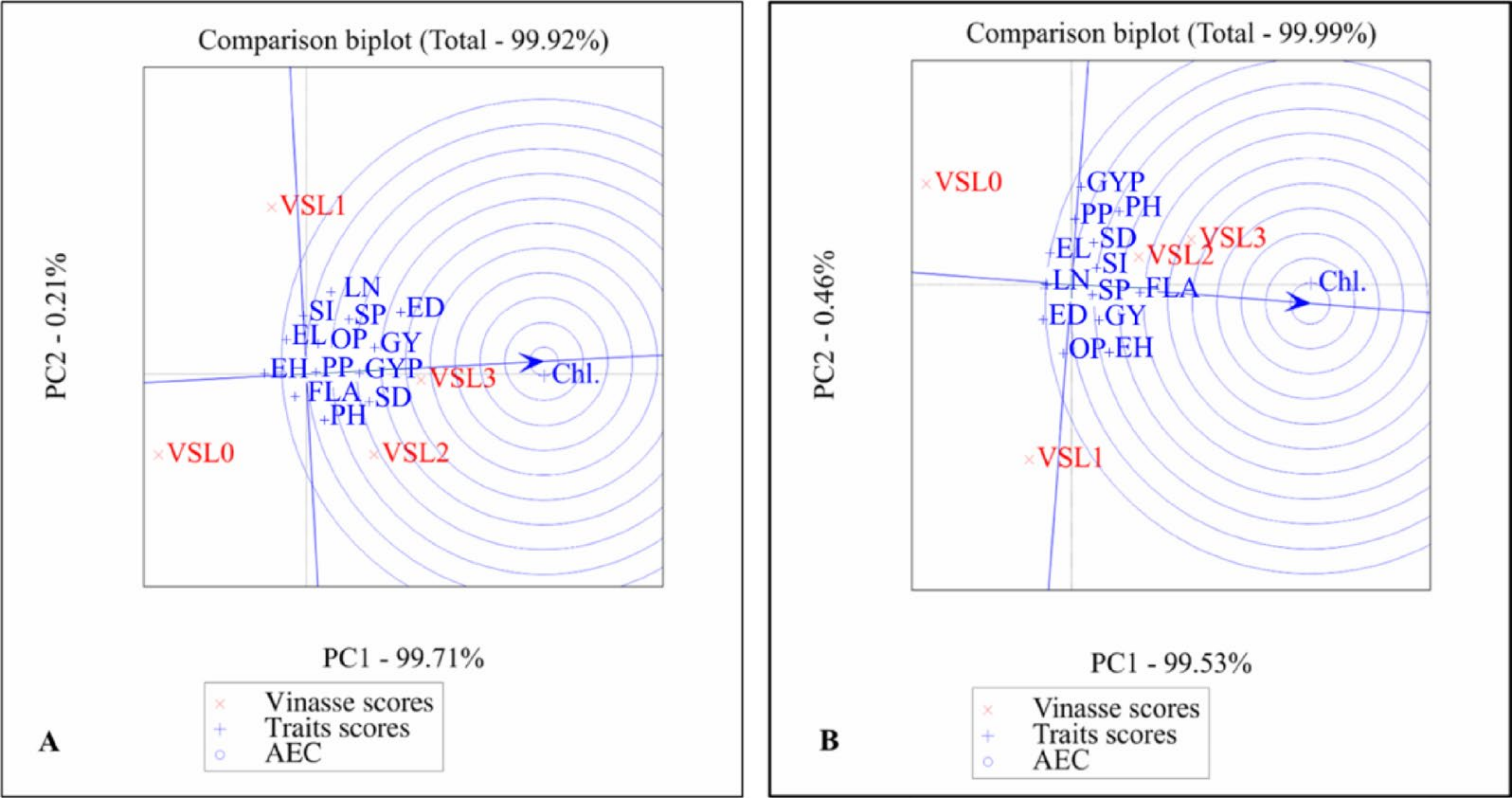
Determination of ideal traits and treatments with GGE biplot on SC2031. PH, plant height; LN, leaf number; FLA, Falge leaf area; Chl., chlorophyll; SD, stem diameter; EH, ear height; ED, ear diameter; EL, ear length; GYP, grains yield/plant; SI, 100-grain weight; SP, sheeling percentage; GY, grain yield; PP, protin percentage; OP, oil percentage.0 until 4 Vinasse. (**A**) (2022) and (**B**) (2023).

The GGE biplot analysis was used to evaluate the interaction between vinasse fertilizer treatments and agronomic traits for Single cross SC2031 during the 2022 (A) and 2023 (B) growing seasons (Fig. 4).

Principal components explained variance PC1 explained over 99.5% of the variance in both years (99.71% in 2022 and 99.53% in 2023), while PC2 accounted for a minimal proportion (0.21% and 0.46% respectively), indicating that most of the variation is captured along the PC1 axis. Trait associations chlorophyll content (Chl.) was strongly associated with PC1 in both years, suggesting it is a key trait influenced by vinasse treatments. It was positioned distinctly from most other traits and vinasse levels, highlighting its unique response pattern.

Vinasse treatment scores vinasse levels (VSL0 to VSL3) were distributed mainly along the PC1 axis, with VSL0 (control) separated from the higher vinasse treatments (VSL2 and VSL3). This separation indicates differential effects of vinasse application rates on trait expression.

Agronomic traits cluster traits such as grain yield per plant (GYP), plant height (PH), and protein percentage (PP) clustered together, showing correlated responses to vinasse treatments, especially at moderate to higher levels. AEC (Average environment coordination) indication the concentric circles centered on the arrow of chlorophyll content represent the ideal trait direction for improvement. The closer a treatment or trait lies to this arrow, the more favorable it is considered in terms of enhancing chlorophyll and related performance indicators.

The GGE biplot highlights the importance of chlorophyll content as an influential trait in response to vinasse fertilization in SC2031. Vinasse levels 2 and 3 demonstrate stronger positive interactions with key yield-related traits, confirming their potential for improving Single cross performance compared to the control (VSL0). The pattern is consistent over both years, suggesting stable responses under vinasse treatments.

#### GGE biplot analysis of vinasse levels and traits for single cross SC2036 (2022 and 2023)

**Fig. 5:**
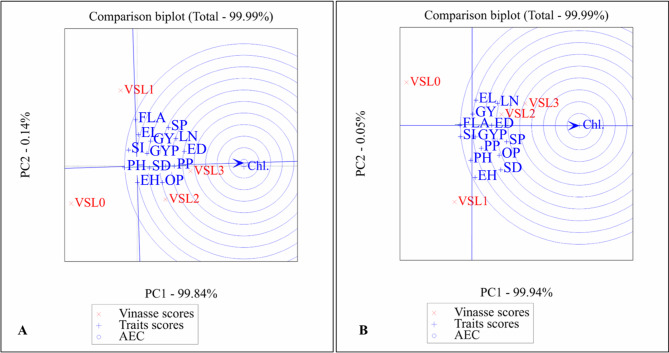
Determination of ideal traits and treatments with GGE biplot on SC2036. PH, plant height; LN, leaf number; FLA, Falge leaf area; Chl., chlorophyll; SD, stem diameter; EH, ear height; ED, ear diameter; EL, ear length; GYP, grains yield/plant; SI, 100-grain weight; SP, sheeling percentage; GY, grain yield; PP, protin percentage; OP, oil percentage. 0 until 4 Vinasse. (**A**) (2022) and (**B**) (2023).

The GGE biplot analysis for single cross SC2036 showed that the first principal component (PC1) captured nearly all variation (> 99%) in both 2022 and 2023, while PC2 contributed minimally. This indicates that most differences in trait responses and vinasse treatment effects are represented along PC1 in (Fig. 5).

Chlorophyll content (Chl.) consistently stood out as a distinctly responsive trait, separated from other agronomic traits and treatments, suggesting it reacts uniquely to vinasse application. Yield-related traits, such as grain yield per plant (GYP) and other growth traits, clustered together, reflecting coordinated responses to vinasse fertilization, particularly at moderate to high levels.

Vinasse treatments VSL2 and VSL3 were closely associated with key agronomic and yield traits, while the control (VSL0) remained separated, indicating limited effectiveness. Overall, higher vinasse levels consistently enhanced crop performance, with chlorophyll content serving as a sensitive indicator of treatment response, demonstrating stable and reproducible effects across both seasons.

#### GGE biplot analysis of vinasse levels and traits for single cross SC168 (2022 and 2023)

**Fig. 6:**
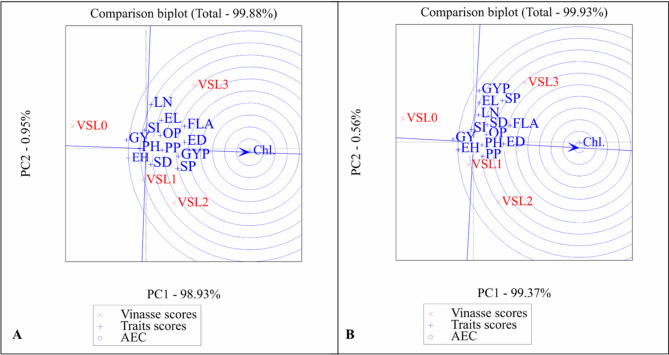
Determination of ideal traits and treatments with GGE biplot on SC168. PH, plant height; LN, leaf number; FLA, Falge leaf area; Chl., chlorophyll; SD, stem diameter; EH, ear height; ED, ear diameter; EL, ear length; GYP, grains yield/plant; SI, 100-grain weight; SP, sheeling percentage; GY, grain yield; PP, protin percentage; OP, oil percentage. 0 until 4 Vinasse. (**A**) (2022) and (**B**) (2023).

The first principal component (PC1) explained **98.93%** of the variation in 2022 and **99.37%** in 2023, while the second component (PC2) contributed **0.95%** and **0.56%**, respectively. This confirms that nearly all meaningful variations are captured along PC1 in (Fig. 6).

Chlorophyll content (Chl.) consistently aligned with PC1 and the ideal vector, highlighting it as a key trait most positively influenced by vinasse treatments. Yield- and growth-related traits, including plant height (PH), ear height (EH), and grain yield per plant (GYP), clustered together, reflecting coordinated and stable responses to vinasse across both seasons.

The GGE biplot results for SC168 indicate that higher vinasse levels, particularly VSL3, are closely associated with improved agronomic performance and photosynthetic efficiency. The control (VSL0) remained separated, showing limited effect. Overall, the findings emphasize the effectiveness of moderate to high vinasse application in enhancing SC168 growth, yield, and stability, with chlorophyll content serving as a reliable indicator of treatment response.

## Discussion

The experiment was conducted over two growing seasons; climatic conditions were largely consistent, with comparable temperature and relative humidity values and no rainfall recorded during either season. Because irrigation was fully controlled and uniformly applied, seasonal environmental variation was minimized. Thus, year-to-year differences in measured traits are more likely attributable to vinasse treatments and genotype responses rather than climatic fluctuations.

The superiority of SC2036 may be attributed to its higher combining ability, wider adaptability, and greater hybrid vigor compared with the other tested single crosses. These characteristics contributed to its improved growth, yield, and grain quality performance. Similar findings were reported by Adhikari et al.^37^. Single cross maize single cross s produces more biomass/plant leading to higher biomass production may be the most relevant cause for higher nutrient uptake. These results agree with Manjhi et al.^38^ who reported that higher plant height, number of leaves plant^−1^, chlorophyll, stem diameter, ear length, ear diameter, grain yield, 100 grain weight and protein and oil percentage were associated with higher fertility of vinasse. The higher growth attributing traits with these fertility level could be owing to increased availability of nutrients. Furthermore, higher yield experienced with single cross s was to higher growth and yield attributes (ear length, ear diameter, 100_grain weight and grain yield/plant. Among the fertility levels of vinasse, the increase in yield at higher fertility levels of vinasse was owing to increased sink capacity. The grain yield of maize mainly depends on the growth and yield attributes. The positive and significant improvements in crop-growth rate and net assimilation rate attributed to higher number of leaves and chlorophyll during crop-growth and development which increased yield attributes resulted in enhanced grain yield. These results are in line with those reported by Kumar et al.^39^. In the other side, increased nutrient levels of vinasse increased the availability of nutrient in the soil, resulting in higher uptake of the nutrients. This enhanced the growth of characters which ultimately increased nutrients concentration in total biomass of plants. These results are in the same line as this obtained Manjhi et al.^38^.

Although the study did not include post-experiment soil analysis, the observed improvements in growth, yield, and quality traits indicate that vinasse application enhanced nutrient availability and crop performance without detectable negative impacts. Future studies are recommended to monitor soil fertility parameters after repeated vinasse applications to fully evaluate long-term sustainability.

The use of correlations between traits commonly known how different traits relate is essential in developing breeding programs to increase grain yield. A negative correlation was observed between shelling percentage and grain yield, likely reflecting a trade-off where higher shelling favors ear structure but slightly limits grain filling. This highlights the need to balance structural and reproductive traits in single cross selection. The correlations indicate that higher leaf number and chlorophyll content enhance maize productivity through improved photosynthesis and biomass partitioning, whereas ear height and shelling percentage reflect trade-offs in resource allocation. These patterns provide useful insights for selecting Single cross s with optimal growth and grain yield in maize breeding programs.

The strong contribution of trait effects to phenotypic variation suggests that these agronomic traits play a key role in determining maize productivity and can be useful indicators for optimizing fertilization strategies under sustainable agricultural systems. These findings are consistent with Yan et al.^40^, who reported that genotype traits often contribute significantly to phenotypic variation when multiple traits are evaluated across different environments.

Although vinasse treatments had a smaller but significant effect on the variation, the interaction between traits and vinasse treatments was also notable. The significant genotype-by-environment interaction (GEI) and the high percentage of interaction variance explained by the first principal component (IPCA1) confirm the complexity of the relationship between genotype performance and vinasse application levels. This is consistent with studies by Gauch and Zobel^41^, Gauch^42^, who emphasized the importance of IPCA1 in capturing most of the GEI in crop trials, making it a critical factor for selecting stable genotypes across environments. The dominance of IPCA1 in explaining GEI variance suggests that specific combinations of traits respond distinctly to vinasse levels, indicating that selecting for traits with positive responses under optimal vinasse treatment can improve overall crop performance. Similar observations were made by Oyekunle et al.^43^, who found that understanding GEI through biplot analysis aids in identifying stable and high-performing genotypes under various soil fertility treatments.

The minimal residual and error sums of squares highlight the suitability of the AMMI model for analyzing the data, confirming its robustness in capturing the main effects and interactions accurately, as supported by Gauch and Zobel^41^. Overall, these results suggest that while vinasse application influences SC 2031 performance, the inherent traits dominate the response pattern. This indicates the potential for breeding programs to focus on traits that maintain stability and yield across vinasse treatment variations. The findings are in line with recommendations by Yan and Tinker^44^, who suggest using AMMI models combined with GGE biplots for comprehensive evaluation of genotype performance and stability in different management conditions. The dominance of vinasse treatments in explaining variability confirms that nutrient management via vinasse significantly affects maize performance, consistent with studies such as Bastos et al.^10^, Sharma^45^, which highlight the critical role of organic amendments on crop growth and productivity. The significant genotype × environment interaction (GEI), particularly captured by IPCA1, suggests that SC2036’s performance is strongly influenced by the interaction of genotype with vinasse treatment levels and environmental factors. This aligns with the findings of Gauch^42^, who emphasize that IPCA1 typically accounts for most GEI variation, making it a useful metric for identifying stability and adaptation patterns. The small contribution from individual traits to the overall variance indicates that while traits are important, their expression is largely conditioned by environmental and treatment factors, as supported by Yan and Rajcan^15^. The effective partitioning of variance by the AMMI model further confirms its suitability for interpreting multi-environment trial data^43^. Overall, these results suggest that vinasse fertilizer management can be optimized to enhance yield and stability in SC2036. The AMMI model’s identification of key interaction components supports targeted breeding and agronomic decisions to improve maize productivity under varying vinasse treatments.

The AMMI analysis highlights the dominant influence of Vinasse fertilizer treatments on the phenotypic traits of SC168 maize single cross, confirming that Vinasse application levels significantly impact growth and yield components, the overwhelming contribution of treatment and traits to the total variance aligns with findings by Gauch^42^, who emphasized the importance of treatment effects in crop performance variance. The significant but limited interaction effects (VF * Traits) and high IPCA 1 value suggest that genotype responses to Vinasse levels are largely consistent, but some specific traits exhibit differential sensitivity. This concurs with results from Momeni et al.^46^, Yan and Rajcan^15^, who documented that the first IPCA typically captures most interaction effects, allowing effective genotype stability assessment. Low IPCA 2 and residual variance imply the AMMI model’s robustness in explaining genotype by environment interactions under Vinasse treatments for SC168. This finding is consistent with studies by Oyekunle et al.^43^, who reported similar patterns in maize single cross s’ response to fertilizer and environmental interactions. These results emphasize the potential for selecting stable single cross s and optimum Vinasse fertilizer rates to maximize maize productivity while maintaining trait stability across years. Such knowledge is crucial for improving fertilizer management strategies and breeding enhanced performance under variable nutrient conditions.

The consistently low ASV values recorded for the single cross SC2031 across different vinasse levels and growing seasons confirm its superior stability and adaptability, indicating reliable performance under varying environmental conditions, which is in agreement with the effectiveness of ASV analysis as reported by Mousavi et al.^47^, Mousavi et al.^48^, Yue et al.^49^, and Alam et al.^50^, and Mousavi et al.^51^. Conversely, the markedly high ASV values observed for SC2036 reflect a strong genotype × environment interaction, indicating high sensitivity and instability in response to vinasse application. Meanwhile, SC168 exhibited moderate stability with a clear improvement during the second season, suggesting a gradual adaptation to the prevailing environmental conditions.

The results suggest that moderate vinasse application can promote both stability and improved performance of SC2031, consistent with previous studies demonstrating the positive effects of organic fertilizers on crop growth and yield^45,52^. The distinct response of the control (VSL0) confirms the impact of vinasse on plant physiology. The wider spread observed in 2022 may reflect seasonal environmental variability or management influences, which aligns with Adhikari et al.^37^, who reported year-to-year differences in fertilizer response.

Traits such as chlorophyll content, which showed weaker associations with vinasse treatments, suggest that vinasse affects growth and yield parameters more strongly than physiological traits—likely because chlorophyll concentration is regulated by multiple factors beyond nutrient availability. Overall, the results support the beneficial use of vinasse as an organic fertilizer to enhance growth and yield traits in maize Single cross SC2031, with the AMMI biplot offering a clear visualization of interaction effects and treatment stability.

These results suggest that moderate vinasse application (VSL1–VSL2) enhances growth and yield traits of SC2036, in agreement with previous studies reporting improved maize performance under organic amendments^53,54^. The weaker association of chlorophyll with vinasse may reflect its multifactorial regulation, influenced by nutrient availability, environmental stress, and genetic factors^55^. Seasonal differences in trait responses likely reflect climatic or soil influences, consistent with observations from other studies on maize-fertilizer interactions^56^. Overall, these findings support the recommendation to apply moderate vinasse levels to maximize productivity in SC2036, promoting sustainable maize production.

Moderate vinasse application (VSL1 and VSL3) enhanced growth and yield traits in SC168, supporting previous studies reporting improved maize vigor under moderate organic fertilizer doses^57,58^. he relatively weak association of chlorophyll with vinasse suggests influence from environmental and genetic factors by Kwon et al.^59^. More consistent trait responses in 2023 may reflect improved environmental conditions or management practices from Sidahmed et al.^60^.

Overall, moderate vinasse levels optimize growth and yield in SC168, promoting sustainable maize production.

The GGE biplot clearly indicates that moderate vinasse application rates (VSL2 and VSL3) enhance key agronomic traits and yield components in Single cross SC2031. This aligns with findings by Iquebal et al.^61^, who reported improved maize biomass and grain yield under moderate organic amendments. The isolated position of chlorophyll content is consistent with previous observations that pigment concentration may not directly correspond to vinasse nutrient supply, but rather to environmental and genetic factors^62^. The clear differentiation of treatments in the biplot suggests that vinasse dosage critically influences trait expression and yield stability, supporting precision nutrient management as advocated by Rose et al.^2^. Overall, the GGE biplot analysis provides a valuable visualization of treatment effects, confirming that vinasse treatments VSL2 and VSL3 are optimal for enhancing growth and yield in Single cross SC2031.

The GGE biplot suggests that moderate vinasse applications (VSL2 and VSL3) optimize growth and yield traits in SC2036, supporting previous studies on the benefits of moderate organic nutrient amendments^63,64^.

The weaker association of chlorophyll with other traits aligns with Mandal et al.^58^, indicating that pigment levels respond differently to nutrient inputs. These findings highlight that precise vinasse management can enhance productivity by promoting desirable traits while avoiding negative effects of excessive or insufficient application^65^.

Moderate vinasse applications (VSL1 and VSL2) optimized growth and yield traits in SC168, consistent with previous studies on nutrient management in maize^66,67^. The distinct response of chlorophyll reflects its sensitivity to nutrients and environmental factors^68^. Divergence of VSL3 and VSL0 treatments indicates reduced efficiency at excessive or absent vinasse application.

Correlation between mean yields and stability parameters confirms that SC2031 was the most stable single cross across seasons, followed by SC2036, while SC168 showed the lowest stability, in agreement with findings by Eberhart & Russell, i.e., Eberhart & Russell^69^, Tai^70^, Tai^71^, Cross^72^, and Eagles et al.^73^, For example, Tai^71^ reported that the high-yielding genotypes were assessable over environments and those possessing average stability were generally low in pro-ductility. Results obtained in the present investigation clearly showed the same conclusion. In this regard, SC2031 represented the most stable single cross across seasons, followed by SC2036, while SC168 showed the lowest stability.

## Conclusion

This study demonstrates that vinasse, when applied at low to moderate doses (VSL1–VSL2), serves as an effective organic fertilizer for enhancing maize productivity. The highest grain yield per plant was obtained with single cross SC2036 under 3 L/plot vinasse, recording 187.0 and 191.2 g in the two seasons. Integration of AMMI and GGE biplot analyses provided robust insights into genotype performance and trait interactions, identifying SC2036 as the most responsive hybrid and SC2031 as the most stable across vinasse treatments and seasons. Chlorophyll content emerged as a key indicator of nutrient efficiency, reinforcing its role in genotype evaluation. These findings support the use of vinasse as a sustainable fertilization strategy in maize cultivation, with further validation recommended across diverse environments and soil conditions.

## Supplementary Information

Supplementary Information 1.

## Data Availability

The data generated from the study will be provided by the corresponding author upon request.
