# Supplementary material for: Sustainable improvement of single cross maize performance using vinasse-based biofertilizer
Source: Sci Rep. 2026 May 13;16:15040. doi: 10.1038/s41598-026-49182-y (PMC13171953; doi:10.1038/s41598-026-49182-y)
Supplement: Supplementary file 1 — Supplementary Information 1. [file 41598_2026_49182_MOESM1_ESM.docx]

Table S1: Correlation analysis of SC2031, SC2036, and SC168 in four Vinasse treatments for 2022(below) and 2023(above diagonal) seasons.

|  | | PH | NL | ALF | Chl. | SD | EH | ED | EL | GYP | SI | SP | GY | PP | OP |
| --- | --- | --- | --- | --- | --- | --- | --- | --- | --- | --- | --- | --- | --- | --- | --- |
| **SC2031** | PH |  | 0.82 | 0.84 | 0.87 | 0.90 | -0.80 | 0.80 | 0.86 | 0.99 | 0.79 | -0.94 | 0.79 | 0.76 | 0.67 |
|  | NL | **0.96** |  | 1.00 | 0.99 | 0.73 | -0.99 | 1.00 | 1.00 | 0.88 | 1.00 | -0.96 | 0.99 | 0.98 | 0.98 |
|  | ALF | **0.97** | **1.00** |  | 0.99 | 0.75 | -0.99 | 1.00 | 1.00 | 0.90 | 1.00 | -0.97 | 0.99 | 0.98 | 0.97 |
|  | Chl. | **0.96** | **0.99** | **1.00** |  | 0.84 | -0.99 | 0.99 | 0.99 | 0.92 | 0.98 | -0.97 | 0.95 | 0.98 | 0.93 |
|  | SD | **0.97** | **0.97** | **0.97** | **0.95** |  | -0.76 | 0.74 | 0.77 | 0.88 | 0.71 | -0.82 | 0.64 | 0.76 | 0.58 |
|  | EH | **-0.96** | **-1.00** | **-1.00** | **-0.99** | **-0.97** |  | -1.00 | -0.99 | -0.86 | -1.00 | 0.95 | -0.97 | -1.00 | -0.97 |
|  | ED | **0.93** | **0.85** | **0.87** | **0.90** | **0.83** | **-0.84** |  | 0.99 | 0.86 | 1.00 | -0.95 | 0.98 | 0.99 | 0.98 |
|  | EL | **0.97** | **0.99** | **0.99** | **0.98** | **0.99** | **-0.99** | **0.83** |  | 0.92 | 0.99 | -0.98 | 0.98 | 0.97 | 0.96 |
|  | GYP | **0.96** | **0.91** | **0.92** | **0.95** | **0.88** | **-0.90** | **0.99** | **0.89** |  | 0.86 | -0.97 | 0.86 | 0.83 | 0.76 |
|  | SI | **0.95** | **1.00** | **1.00** | **0.99** | **0.97** | **-1.00** | **0.83** | **0.99** | **0.90** |  | -0.95 | 0.99 | 0.99 | 0.98 |
|  | SP | **-0.91** | **-0.95** | **-0.95** | **-0.91** | **-0.98** | **0.96** | **-0.71** | **-0.98** | **-0.78** | **-0.96** |  | -0.95 | -0.92 | -0.89 |
|  | GY | **0.97** | **1.00** | **1.00** | **0.99** | **0.98** | **-1.00** | **0.85** | **1.00** | **0.91** | **1.00** | **-0.96** |  | 0.95 | 0.98 |
|  | PP | **0.93** | **1.00** | **0.99** | **0.99** | **0.95** | **-1.00** | **0.81** | **0.98** | **0.88** | **1.00** | **-0.95** | **0.99** |  | 0.97 |
|  | OP | **0.88** | **0.96** | **0.95** | **0.92** | **0.95** | **-0.97** | **0.68** | **0.97** | **0.76** | **0.97** | **-0.99** | **0.96** | **0.97** |  |
| **SC2036** | PH |  | 0.94 | 0.97 | 0.99 | 0.96 | -1.00 | 0.98 | 0.98 | 0.98 | 0.95 | -0.97 | 0.91 | 1.00 | 0.91 |
|  | NL | **0.96** |  | 0.97 | 0.97 | 0.97 | -0.95 | 0.99 | 0.97 | 0.98 | 0.98 | -0.83 | 0.82 | 0.92 | 1.00 |
|  | ALF | **0.98** | **0.98** |  | 0.98 | 1.00 | -0.97 | 0.99 | 0.95 | 0.99 | 1.00 | -0.92 | 0.93 | 0.96 | 0.96 |
|  | Chl. | **1.00** | **0.97** | **0.99** |  | 0.97 | -1.00 | 0.99 | 0.99 | 1.00 | 0.97 | -0.92 | 0.88 | 0.98 | 0.95 |
|  | SD | **0.94** | **0.93** | **0.98** | **0.95** |  | -0.96 | 0.98 | 0.94 | 0.99 | 1.00 | -0.92 | 0.94 | 0.96 | 0.95 |
|  | EH | **-1.00** | **-0.96** | **-0.98** | **-1.00** | **-0.94** |  | -0.98 | -0.99 | -0.99 | -0.95 | 0.95 | -0.89 | -0.99 | -0.92 |
|  | ED | **0.94** | **0.82** | **0.91** | **0.94** | **0.91** | **-0.95** |  | 0.99 | 1.00 | 0.98 | -0.90 | 0.88 | 0.96 | 0.98 |
|  | EL | **0.96** | **0.89** | **0.97** | **0.96** | **0.98** | **-0.96** | **0.97** |  | 0.98 | 0.94 | -0.89 | 0.82 | 0.96 | 0.95 |
|  | GYP | **0.95** | **0.84** | **0.93** | **0.95** | **0.93** | **-0.95** | **1.00** | **0.99** |  | 0.99 | -0.92 | 0.90 | 0.97 | 0.97 |
|  | SI | **0.94** | **0.98** | **0.99** | **0.96** | **0.97** | **-0.95** | **0.84** | **0.93** | **0.87** |  | -0.90 | 0.92 | 0.94 | 0.97 |
|  | SP | **-0.99** | **-0.93** | **-0.94** | **-0.98** | **-0.88** | **0.99** | **-0.93** | **-0.91** | **-0.93** | **-0.89** |  | -0.97 | -0.98 | -0.79 |
|  | GY | **0.99** | **0.93** | **0.98** | **0.99** | **0.97** | **-0.99** | **0.97** | **0.99** | **0.98** | **0.94** | **-0.96** |  | 0.94 | 0.79 |
|  | PP | **0.99** | **0.99** | **0.99** | **0.99** | **0.95** | **-0.99** | **0.89** | **0.94** | **0.91** | **0.98** | **-0.96** | **0.97** |  | 0.88 |
|  | OP | **0.93** | **0.99** | **0.97** | **0.94** | **0.93** | **-0.93** | **0.78** | **0.87** | **0.81** | **0.99** | **-0.89** | **0.91** | **0.98** |  |
| **SC168** | PH |  | 0.85 | 0.93 | 0.99 | 0.98 | -0.86 | 0.96 | 0.95 | 0.87 | 0.96 | -0.99 | 0.89 | 0.83 | 0.85 |
|  | NL | **0.98** |  | 0.93 | 0.91 | 0.91 | -0.99 | 0.95 | 0.96 | 1.00 | 0.92 | -0.81 | 0.99 | 0.91 | 0.93 |
|  | ALF | **0.92** | **0.98** |  | 0.97 | 0.98 | -0.89 | 0.99 | 0.99 | 0.96 | 0.99 | -0.92 | 0.98 | 0.98 | 0.98 |
|  | Chl. | **0.97** | **0.99** | **0.96** |  | 1.00 | -0.90 | 0.99 | 0.98 | 0.92 | 0.99 | -0.98 | 0.94 | 0.89 | 0.91 |
|  | SD | **0.85** | **0.91** | **0.87** | **0.95** |  | -0.89 | 0.99 | 0.99 | 0.93 | 1.00 | -0.98 | 0.95 | 0.92 | 0.93 |
|  | EH | **-0.93** | **-0.92** | **-0.92** | **-0.87** | **-0.67** |  | -0.94 | -0.93 | -0.97 | -0.89 | 0.81 | -0.96 | -0.83 | -0.87 |
|  | ED | **0.92** | **0.97** | **1.00** | **0.95** | **0.86** | **-0.92** |  | 1.00 | 0.97 | 0.99 | -0.94 | 0.98 | 0.93 | 0.95 |
|  | EL | **0.94** | **0.97** | **0.99** | **0.94** | **0.82** | **-0.96** | **0.99** |  | 0.97 | 0.99 | -0.93 | 0.99 | 0.95 | 0.97 |
|  | GYP | **0.92** | **0.96** | **0.99** | **0.92** | **0.79** | **-0.96** | **0.99** | **1.00** |  | 0.94 | -0.83 | 1.00 | 0.94 | 0.96 |
|  | SI | **0.96** | **0.99** | **0.99** | **0.97** | **0.86** | **-0.95** | **0.99** | **1.00** | **0.99** |  | -0.96 | 0.96 | 0.95 | 0.96 |
|  | SP | **-0.97** | **-1.00** | **-0.99** | **-0.98** | **-0.89** | **0.93** | **-0.99** | **-0.99** | **-0.98** | **-1.00** |  | -0.86 | -0.83 | -0.84 |
|  | GY | **0.95** | **0.98** | **0.98** | **0.95** | **0.81** | **-0.97** | **0.99** | **1.00** | **1.00** | **1.00** | **-0.99** |  | 0.96 | 0.98 |
|  | PP | **0.86** | **0.93** | **0.99** | **0.89** | **0.79** | **-0.91** | **0.99** | **0.98** | **0.99** | **0.96** | **-0.95** | **0.97** |  | 1.00 |
|  | OP | **0.89** | **0.96** | **1** | **0.936** | **0.86** | **-0.90** | **0.998** | **0.99** | **0.98** | **0.98** | **-0.97** | **0.97** | **0.99** |  |
| Plant Height (PH), Leaf Number (LN), Falge leaf area (FLA), Chlorophyll (Chl.), Stem Diameter (SD), Ear Height (EH), Ear Diameter (ED), Ear Length (EL), Grains Yield Plant^-1^ (GYP), 100-Grain Weight (SI), Sheeling Percentage (SP), Grain Yield/ plot (GY), Protin Percentage (PP), Oil Percentage (OP). | | | | | | | | | | | | | | | |

| Table S2: AMMI Analysis of Variance for SC2031 under Different Vinasse Treatments across Two Successive Seasons (2022 and 2023). | | | | | | | | |
| --- | --- | --- | --- | --- | --- | --- | --- | --- |
| S.O. V | D.F | | SS | | SS% |  | F | |
| Year |  |  | **2022** | **2023** | **2022** | **2023** | **2022** | **2023** |
| Total | 167 | | 2360230.0 | 2527002.0 |  |  |  |  |
| Treatments | 55 | | 2359475.0 | 2526359.0 |  |  | 42394.8 | 14783.3 |
| Vinasse | 3 | | 7105.0 | 6622.0 |  |  | 2340.5 | 710.4 |
| Traits | 13 | | 2287712.0 | 2454725.0 |  |  | 173907.5 | 60771.3 |
| Block | 28 | | 670.0 | 382.0 |  |  | 23.6 | 4.4 |
| Interactions | 39 | | 64657.0 | 65013.0 |  |  | 1638.4 | 536.5 |
| IPCA 1 | 15 | | 64475.0 | 64716.0 | 99.72 | 99.54 | 4247.8 | 1388.5 |
| IPCA 2 | 13 | | 127.0 | 288.0 | 0.20 | 0.44 | 9.7 | 7.1 |
| Residuals | 11 | | 56.0 | 8.0 | 0.09 | 0.01 | 5.0 | 0.2 |
| Error | 84 | | 85.0 | 572.0 |  |  |  |  |

| Table S3: AMMI Analysis of Variance for SC2036 under Different Vinasse Treatments across Two Successive Seasons (2022 and 2023). | | | | | | | | |
| --- | --- | --- | --- | --- | --- | --- | --- | --- |
| S.O. V | D.F | | SS | | SS% |  | F | |
| Year |  |  | **2022** | **2023** | **2022** | **2023** | **2022** | **2023** |
| Total | 167 | | 2420079.0 | 2577011.0 |  |  |  |  |
| Vinasse | 55 | | 2419516.0 | 2576320.0 |  |  | 25661.5 | 6072.1 |
| Traits | 3 | | 7065.0 | 6239.0 |  |  | 1373.8 | 269.6 |
| Environments | 13 | | 2349093.0 | 2506372.0 |  |  | 105408.0 | 24992.3 |
| Block | 28 | | 418.0 | 43.0 |  |  | 8.7 | 0.2 |
| Interactions | 39 | | 63359.0 | 63709.0 |  |  | 947.7 | 211.8 |
| IPCA 1 | 15 | | 63258.0 | 63670.0 | 99.84 | 99.94 | 2460.0 | 550.2 |
| IPCA 2 | 13 | | 92.0 | 33.0 | 0.15 | 0.05 | 4.1 | 0.3 |
| Residuals | 11 | | 9.0 | 5.0 | 0.01 | 0.01 | 0.5 | 0.1 |
| Error | 84 | | 144.0 | 648.0 |  |  |  |  |

| Table S4: AMMI Analysis of Variance for SC168 under Different Vinasse Treatments across Two Successive Seasons (2022 and 2023). | | | | | | | | |
| --- | --- | --- | --- | --- | --- | --- | --- | --- |
| S.O.V | d.f. | | SS | | SS% |  | F | |
| Year |  |  | **2022** | **2023** | **2022** | **2023** | **2022** | **2023** |
| Total | 167 | | 1826511.0 | 1950223.0 |  |  |  |  |
| Treatments | 55 | | 1825974.0 | 1949891.0 |  |  | 126763.6 | 48032.5 |
| Vinasse | 3 | | 1876.0 | 2507.0 |  |  | 2386.4 | 1132.2 |
| Traits | 13 | | 1805914.0 | 1924861.0 |  |  | 530406.5 | 200605.9 |
| Block | 28 | | 515.0 | 270.0 |  |  | 68.7 | 13.1 |
| Interactions | 39 | | 18184.0 | 22523.0 |  |  | 1779.3 | 782.4 |
| IPCA 1 | 15 | | 17973.0 | 22378.0 | 98.84 | 99.4 | 4574.2 | 2021.2 |
| IPCA 2 | 13 | | 188.0 | 131.0 | 1.03 | 0.58 | 53.5 | 13.7 |
| Residuals | 11 | | 23.0 | 14.0 | 0.13 | 0.1 | 7.6 | 1.7 |
| Error | 84 | | 22.0 | 62.0 |  |  |  |  |

| Table S5: Values of both IPCA1 and IPCA2 for each single cross of maize across measured traits. | | | | | | | | | | | | |
| --- | --- | --- | --- | --- | --- | --- | --- | --- | --- | --- | --- | --- |
| Trait means and scores | SC2031 | | | | SC2036 | | | | SC168 | | | |
|  | 2022 | | 2023 | | 2022 | | 2023 | | 2022 | | 2023 | |
|  | IPCA1 | IPCA2 | IPCA1 | IPCA2 | IPCA1 | IPCA2 | IPCA1 | IPCA2 | IPCA1 | IPCA2 | IPCA1 | IPCA2 |
| FLA | 0.71 | -0.37 | 0.71 | -0.37 | 1.20 | -0.47 | 1.13 | -0.68 | 1.36 | 0.19 | 1.20 | 0.25 |
| Chl. | -8.67 | -0.30 | -8.67 | -0.30 | -11.34 | 0.23 | -11.45 | 0.22 | -11.42 | 0.35 | -11.49 | 0.29 |
| ED | 0.77 | 0.20 | 0.77 | 0.20 | 1.00 | 0.04 | 0.94 | 0.04 | 1.01 | 0.16 | 0.96 | 0.37 |
| EH | 2.24 | -2.13 | 2.24 | -2.13 | 2.48 | 0.33 | 2.33 | 0.75 | 2.37 | -0.11 | 2.37 | -0.31 |
| EL | 0.59 | 0.29 | 0.59 | 0.29 | 0.94 | 0.03 | 0.85 | 0.05 | 0.91 | 0.30 | 0.86 | 0.34 |
| GY | 0.73 | 0.28 | 0.73 | 0.28 | 0.99 | 0.13 | 0.94 | -0.15 | 1.01 | 0.31 | 0.98 | 0.35 |
| GYP | 0.25 | 0.95 | 0.25 | 0.95 | -0.26 | -2.12 | 0.36 | 0.12 | -0.26 | -1.69 | 0.03 | -1.34 |
| NL | 0.65 | 0.45 | 0.65 | 0.45 | 0.81 | 0.52 | 0.74 | 0.39 | 0.76 | 0.40 | 0.75 | 0.43 |
| OP | 0.77 | 0.22 | 0.77 | 0.22 | 1.03 | 0.25 | 0.96 | 0.09 | 1.03 | 0.35 | 0.99 | 0.39 |
| PH | -0.91 | -0.51 | -0.91 | -0.51 | -0.74 | 0.04 | -0.46 | -1.35 | -0.59 | -1.59 | -0.26 | -2.50 |
| PP | 0.70 | 0.24 | 0.70 | 0.24 | 0.99 | 0.23 | 0.93 | -0.10 | 0.98 | 0.36 | 0.93 | 0.41 |
| SD | 0.78 | 0.17 | 0.78 | 0.17 | 1.01 | 0.16 | 0.95 | 0.01 | 1.02 | 0.28 | 1.00 | 0.29 |
| SI | 0.34 | 0.17 | 0.34 | 0.17 | 0.73 | 0.45 | 0.62 | 0.19 | 0.64 | 0.45 | 0.54 | 0.60 |
| SP | 1.06 | 0.35 | 1.06 | 0.35 | 1.15 | 0.19 | 1.17 | 0.42 | 1.18 | 0.25 | 1.16 | 0.43 |
| Falge leaf area (FLA), Chlorophyll (Chl.), Ear Diameter (ED), Ear Height (EH), Ear Length (EL), Grain Yield/ plot (GY), Grains Yield Plant-1 (GYP),Number of leaves/ plant (NL), Oil Percentage (OP),Plant Height (PH), Protin Percentage (PP),Stem Diameter (SD), 100-Grain Weight (SI), Sheeling Percentage (SP). | | | | | | | | | | | | |

| Table S6:. Values of both IPCA1 and IPCA2 for each single cross of maize across vinasse levels. | | | | | | | | | | | | |
| --- | --- | --- | --- | --- | --- | --- | --- | --- | --- | --- | --- | --- |
| vinase level. means and scores | SC2031 | | | | SC2036 | | | | SC168 | | | |
|  | 2022 | | 2023 | | 2022 | | 2023 | | 2022 | | 2023 | |
|  | IPCA1 | IPCA2 | IPCA1 | IPCA2 | IPCA1 | IPCA2 | IPCA1 | IPCA2 | IPCA1 | IPCA2 | IPCA1 | IPCA2 |
| VSL0 | 6.98 | 0.70 | 7.22 | 0.71 | 8.70 | 0.99 | 8.67 | 0.87 | 8.83 | 1.02 | 8.56 | 1.54 |
| VSL1 | 0.32 | -0.97 | 0.54 | -0.74 | 2.19 | -1.97 | 2.31 | -1.53 | 2.13 | -2.09 | 2.58 | -2.61 |
| VSL2 | -2.66 | -1.66 | -2.49 | -1.65 | -3.81 | 0.80 | -3.93 | 0.22 | -4.08 | 1.04 | -4.01 | 0.38 |
| VSL3 | -4.64 | 1.93 | -5.27 | 1.68 | -7.08 | 0.18 | -7.05 | 0.44 | -6.88 | 0.04 | -7.13 | 0.69 |
| Vinasse solution plus: The applied rates of a control 0 L/plot (VSL0), 1 L/plot (VSL1), 2 L/plot (VSL2), and 3 L/plot (VSL3) (10.5 m²) | | | | | | | | | | | | |
